# Bioinspired soft electroreceptors for artificial precontact somatosensation

**DOI:** 10.1126/sciadv.abo5201

**Published:** 2022-05-27

**Authors:** Zi Hao Guo, Hai Lu Wang, Jiajia Shao, Yangshi Shao, Luyao Jia, Longwei Li, Xiong Pu, Zhong Lin Wang

**Affiliations:** 1 Beijing Institute of Nanoenergy and Nanosystems, Chinese Academy of Sciences, Beijing 100083, P. R. China.; 2 School of Nanoscience and Technology, University of Chinese Academy of Sciences, Beijing 100049, P. R. China.; 3 Center on Nanoenergy Research, School of Physical Science and Technology, GuangXi University, Nanning 530004, P. R. China.; 4 School of Materials Science and Engineering, Georgia Institute of Technology, Atlanta, GA 30332, USA.

## Abstract

Artificial haptic sensors form the basis of touch-based human-interfaced applications. However, they are unable to respond to remote events before physical contact. Some elasmobranch fishes, such as seawater sharks, use electroreception somatosensory system for remote environmental perception. Inspired by this ability, we design a soft artificial electroreceptor for sensing approaching targets. The electroreceptor, enabled by an elastomeric electret, is capable of encoding environmental precontact information into a series of voltage pulses functioning as unique precontact human interfaces. Electroceptor applications are demonstrated in a prewarning system, robotic control, game operation, and three-dimensional object recognition. These capabilities in perceiving proximal precontact events can lenrich the functionalities and applications of human-interfaced electronics.

## Teaser

Bioinspired soft electroreceptors are reported for precontact human-machine interface and target profile recognition.

## INTRODUCTION

Biological systems provide ideal templates for state-of-the-art bionic electronics, typified by humanoid robotics (*1*–*3*), artificial receptors (*4*, *5*), actuators (*6*), and prosthetics (*7*, *8*). Analogous to mechanoreceptors of the skin, haptic sensors that are highly responsive to mechanical stimuli from surroundings have been deployed in various applications of autonomous devices (*9*–*11*), human-machine interfaces (HMIs) (*12*–*14*), and virtual/augmented reality (*15*, *16*). Nevertheless, haptic sensors operate only when they are physically touched or invaded (*17*) but fail to respond to precontact stimuli. Therefore, it is vital to develop advanced sensors that can detect proximal precontact events, which requires the development of reliable sensing systems beyond the contact-mode paradigm (*18*, *19*). Precontact probing, however, remains challenging, as it involves weak signals that need to be detected accurately within a practical distance (*20*).

Previous work has demonstrated precontact or noncontact detection based on light-sensing elements (*21*), electromagnetic technologies (*22*), or ultrasound transducers (*23*). These strategies generally rely on perceiving infrared radiation or visible spectrum, whereas they are severely affected by environmental parameters, such as ambient light (*24*), weather variations (*25*), and temperature (*26*). Besides, with the progresses of soft robotics (*27*) and stretchable electronics (*28*), future touchless sensors or interfaces are also required with sufficient flexibility or stretchability.

**Fig. 1.**
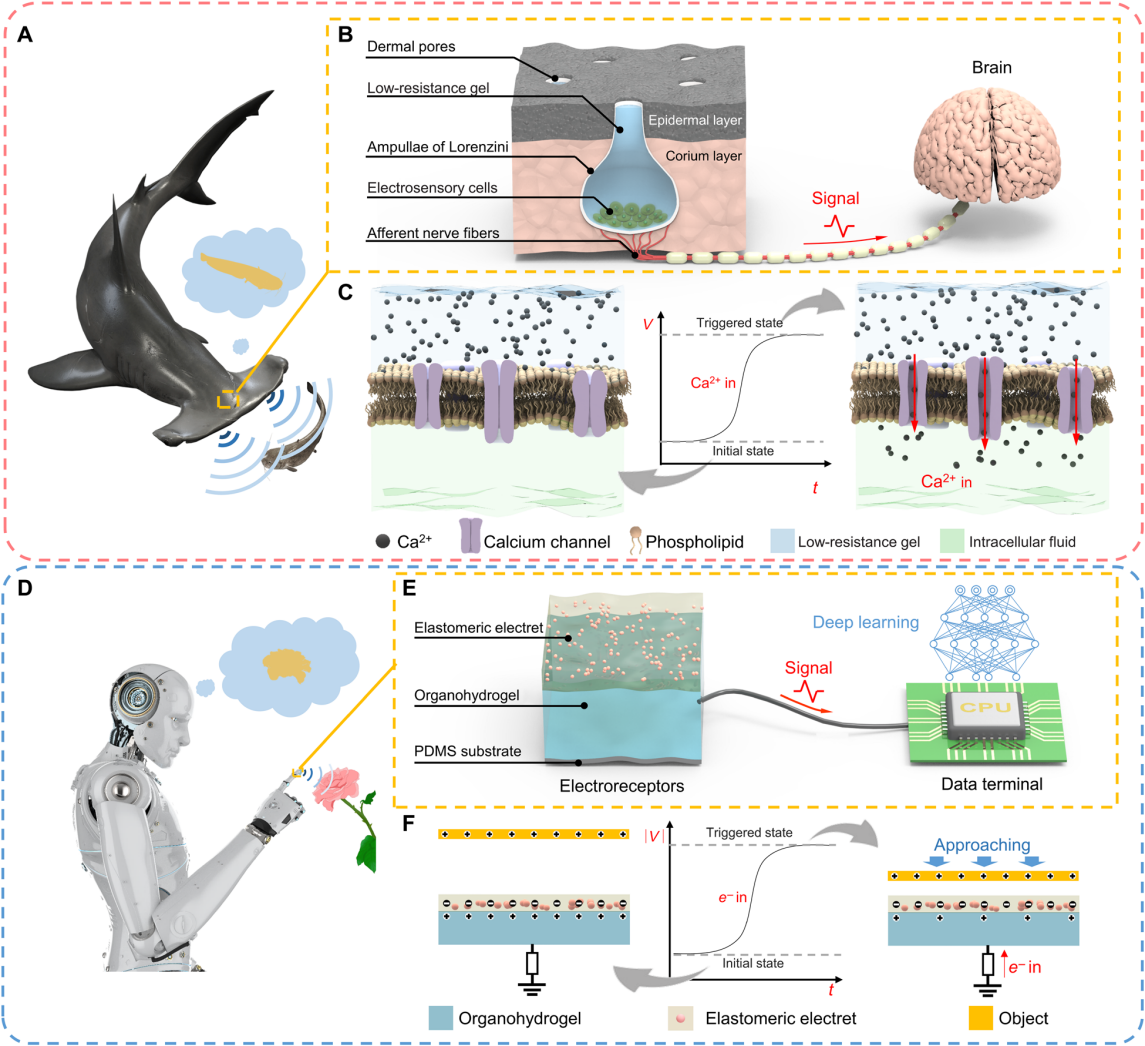
Bioinspired soft electroreceptor. (**A**) Schematic demonstration of the electrosensory system that is distributed on the shark’s head for environmental perception. (**B**) The structure of the shark’s electroreceptor. (**C**) The shark’s sensing strategies. (**D**) Schematic demonstration of the bioinspired soft artificial electroreceptor that is integrated on a robot’s finger for target perception. (**E**) The structure of the artificial electroreceptor. (**F**) The sensing mechanism of the artificial electroreceptor.

Intriguingly, some nature organisms, such as sharks in the dark sea, use an electroreception strategy for remote perception (*29*). A shark can track minute electric field gradients from ambient environment through a mass of electroreceptors distributed on its head (Fig. 1A). Specifically, electric signals are detected by dermal pores and transmitted to shark’s electrosensory cells, and then the electrically gated ion channels will be opened, allowing migration of ions across the cell membrane (Fig. 1, B and C) (*30*). This process leads to the upswing in cell membrane potential, and this variation in membrane potential transmit to the brain as pulse signals, which help the sharks to detect, communicate, prey, and navigate (*31*).

Inspired by the shark’s electrosensory system, we have engineered a flexible transparent artificial electroreceptor using durable and biocompatible components (Fig. 1D). This artificial electroreceptor is capable of sensing approaching targets by detecting the charges that they carry naturally. The electroreceptor adopts a single-electrode configuration; the architecture (Fig. 1E) comprises a layer of thermally charged elastomeric electret, a layer of conductive organohydrogel acting as the ionic electrode, and a layer of encapsulating silicone substrate [polydimethylsiloxane (PDMS)]. Enabled by the electrostatic induction effect, the artificial electroreceptor is able to generate considerable electrical outputs when a naturally charged external target is approaching (fig. S1 and text S1). On this basis, versatile touchless HMIs are designed to orientate targets, manipulate robot arms, and play computer games successfully. Assisted by machine learning algorithms, it also demonstrated the feasibility of the artificial electroreceptor matrix in distinguishing the surface profiles of the targets. Therefore, this work provides insights into sophisticated and multidimensional environmental perception using soft devices, which may find wide applications in wearable devices, soft robotics, smart prosthetics, augmented reality, and so on.

## RESULTS

### Design and working mechanism of the artificial electroreceptor

The organohydrogel in this study is a hybrid polyacrylamide gel that contains ethylene glycol (EG) and water as solvents. The ionic conductivity of the organohydrogel electrode was measured to be 0.845 S/m due to the participation of LiCl. The electret film adopts a composite structure by embedding inorganic electret nanoparticles (SiO_2_) into the dielectric matrix of PDMS elastomers, followed by a modified thermal charging treatment (*32*). Detailed experimental procedures and characterization are described in the Supplementary Materials (figs. S2 and S3). Compared with other fillers, SiO_2_ nanoparticles endow the composite electret with the highest surface charge density (fig. S4). This composite electret enables two functions, where the inorganic nanoparticles trap the charges effectively and the silicone elastomer enables the stretchability of the film. Mechanical testing data show desirable stretchability of the electroreceptor, which can accommodate a tensile strain that exceeds 190% (fig. S5). Besides, the stress-strain curves of the electroreceptor and the electret layer are generally consistent, whereas the elastic modulus of the organohydrogel is three orders of magnitude lower, thus enabling the electroreceptor to stretch freely without delaminating.

On the basis of electrostatics, our electroreceptor can encode environmental precontact information into voltage pulses to achieve proximal sensing (Fig. 1F). Specifically, the electret is negatively precharged initially, and cations accumulate in the ionic electrode layer underneath to balance the static charges (fig. S6A). Meanwhile, the same amount of anions is formed in the electrical double layer at the interface between the metal wire/ionic electrode. When a target with surface charges (positive charges for illustration) approaches the electroreceptor, the cation amount on the surface of ionic electrode will drop because of electrostatic induction mechanism, and so does the anions at the interface between the metal wire/electrode (*33*). This results in electron transfers from the ground to the metal wire through the external circuit, thus outputting voltage pulses (*V*_O_). Likewise, if the targets carry negative surface charges, then the overall process will be reversed, and a flow of electrons will transfer from the metal/ionic electrode interface to the ground (fig. S6B). Therefore, measuring the voltages across an external load allows the precontact detection of the targets.

**Fig. 2.**
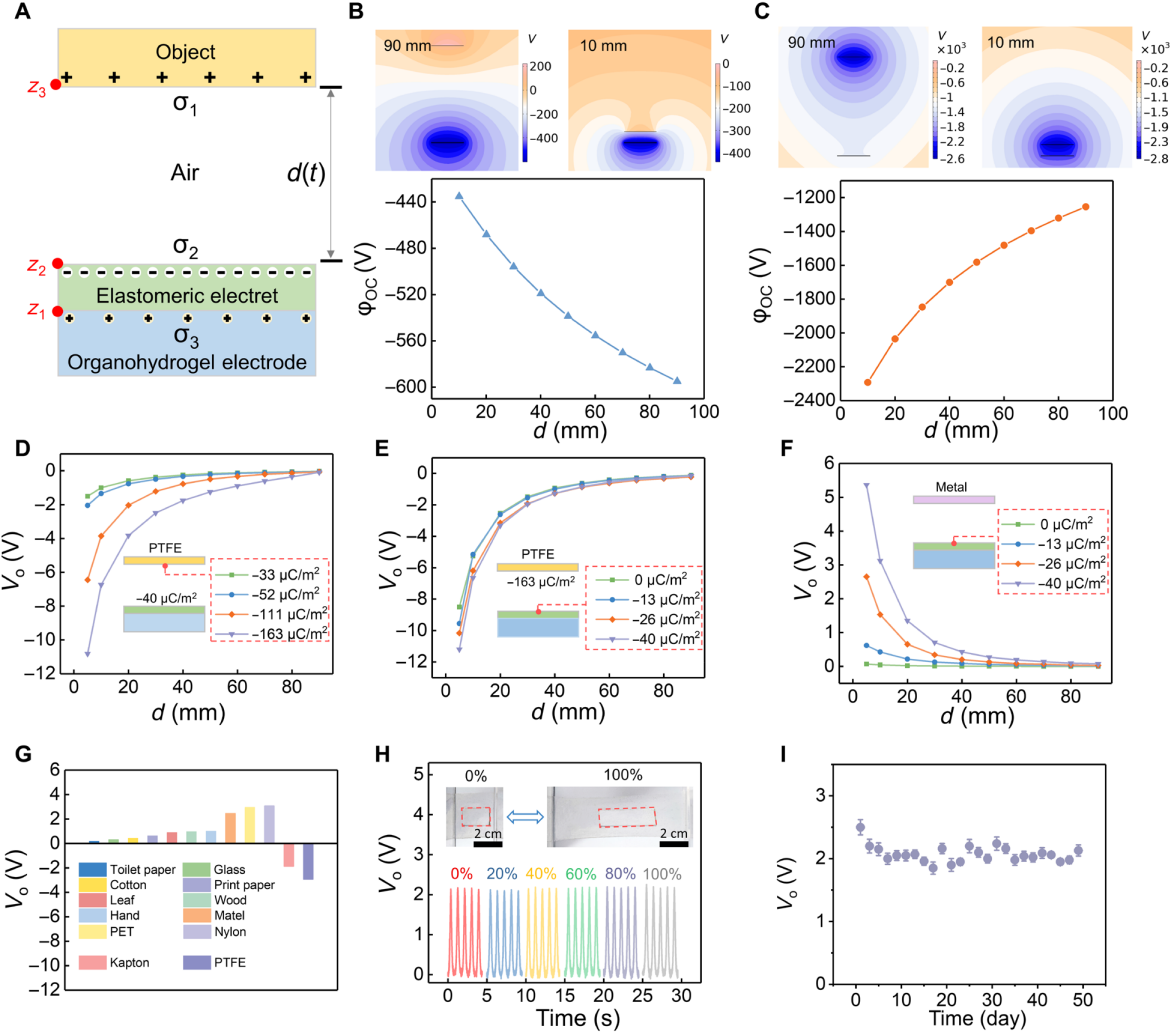
The characteristics of the electroreceptor for precontact sensing. (**A**) The simplified physical model of the electroreceptor. The simulated outputs of the electroreceptor when (**B**) a metal or (**C**) a charged polytetrafluoroethylene (PTFE) approaches, respectively. The relationships between output voltage of the electroreceptor and (**D**) the surface charge density σ_1_ of the target and (**E**) the surface charge density of elastomeric electret σ_2_ when the PTFE film is selected as sensing target experimentally. (**F**) The relationships between the output voltage of the electroreceptor and the surface charge density σ_2_ of electret film when a metal is the sensing target. (**G**) The applicability of the electroreceptor for different materials. (**H**) The output of the electroreceptor under the stretched conditions. (**I**) Durability test of the electroreceptor.

Detailed theoretical explanation of the electroreceptor behavior is provided by mathematical analytics and finite element method simulation (fig. S1; see text S1 for details). Electric output process is described using a simplified physical model in Fig. 2A, and ϕ_OC_ (the electric potential of the ionic electrode) is used to characterize the theoretical performance of the electroreceptor. The variation of simulated ϕ_OC_ was plotted when a metal film (Fig. 2B) and a negatively charged object (Fig. 2C) approaches the elastomeric electret, respectively (movie S1). The metal surface will be inductively charged with positive charges when approaching the electret, which accounts for the reverse ϕ_OC_ variation trend comparing with negatively charged object. Nevertheless, despite the charge signs of the approaching surfaces, the absolute value |∆ϕ_OC_| increases markedly when decreasing the gap distance.

### Output characteristics of the artificial electroreceptor

Then, the sensing capability of the electroreceptor was tested with gap distances ranging from 5 to 90 mm, indicating that the measured voltages (*V*_O_) closely traced the position of the target (Fig. 2, D to F). The effects of charge densities in the approaching target surfaces (σ_1_) and the electret (σ_2_) are obvious. Two characteristics can be summarized: (i) For an insulating but charged approaching target, i.e., a polytetrafluoroethylene (PTFE) film in Fig. 2 (D and E), the distance sensitivity and absolute value of output voltage increase markedly with the charge density σ_1_ of the approaching surface but are insensitive to the charge density of the electret σ_2_, as σ_1_ is independent on σ_2_ in this case; and (ii) for an approaching metal target, the distance sensitivity increase with the electret charge density σ_2_ (Fig. 2F and movie S1), as the induced charges on the metal surface are dependent on the electret charges. These characteristics are consistent with simulation results (fig. S7). In addition, it is observed that the sensing ability is positively proportional to the charge density, and the voltage will be undetectable beyond a certain distance. Therefore, our soft electroreceptor was confirmed to be suitable as a proximal but not a remote precontact sensor. The practical viability of the electroreceptor is also based on the following optimizations: (i) Greater electret charge density is beneficial to the output voltage and the sensitivity of the electroreceptor. In this study, we conducted a series of experiments to optimize the thermal charging parameters; charge density of the electret was optimized at 40 μC/m^2^ (fig. S8), and all the following electrets were fabricated with the optimized condition if without other description. (ii) The electroreceptor is able to detect both metals and nonmetal dielectrics that carry surface charges. Ideally, most of the objects in our daily life carry charges naturally due to contact electrification. They can be charged not only when coming into contact with other types of materials but also when they contact with the same type of material that they are made of (*34*–*36*). Therefore, the electroreceptor is capable of detecting the vast majority of targets theoretically, and this expectation is verified by results in Fig. 2G. We experimented 12 kinds of materials in our laboratory without further treatment, which cover glass, metal, polymer, plastic, and natural material, and all targets were sensed successfully. In addition, output voltage of the electroreceptor shows no substantial decrease under stretched states (up to 100%), compatible with demands for flexible and stretchable devices (Fig. 2H). The elastomeric electret and the organohydrogel did not detach during the 100% stretching process (fig. S9), although no further treatment was designed to improve the interface adhesion. It is noted that the interface adhesion can also be improved according to our previous study (*37*). The performance of the electroreceptor remained stable throughout 50 days of storing at room temperature, confirming also the charge stability of the elastomeric electret (Fig. 2I). We attribute this stability to two aspects: the ability of the electret to preserve the electrostatic charges for longevity, and the solvents of organohydrogel electrodes are not subject to leakage or evaporation under room temperature (fig. S10) (*33*, *38*). In detail, the EG/water binary system, the polarity of Li^+^, and the encapsulation of electrode all worked together to significantly alleviate the water evaporation, contributing to the stability of organohydrogel electrode. Further improvement of stability could be achieved by replacing the organohydrogel electrode to dry ion-conducting elastomers (*38*). In addition, we compared the output of the electroreceptor under different environmental conditions (light and darkness and various temperatures). The results were independent on ambient brightness (fig. S11) and certain temperature range (fig. S12).

### Touchless HMI

**Fig. 3.**
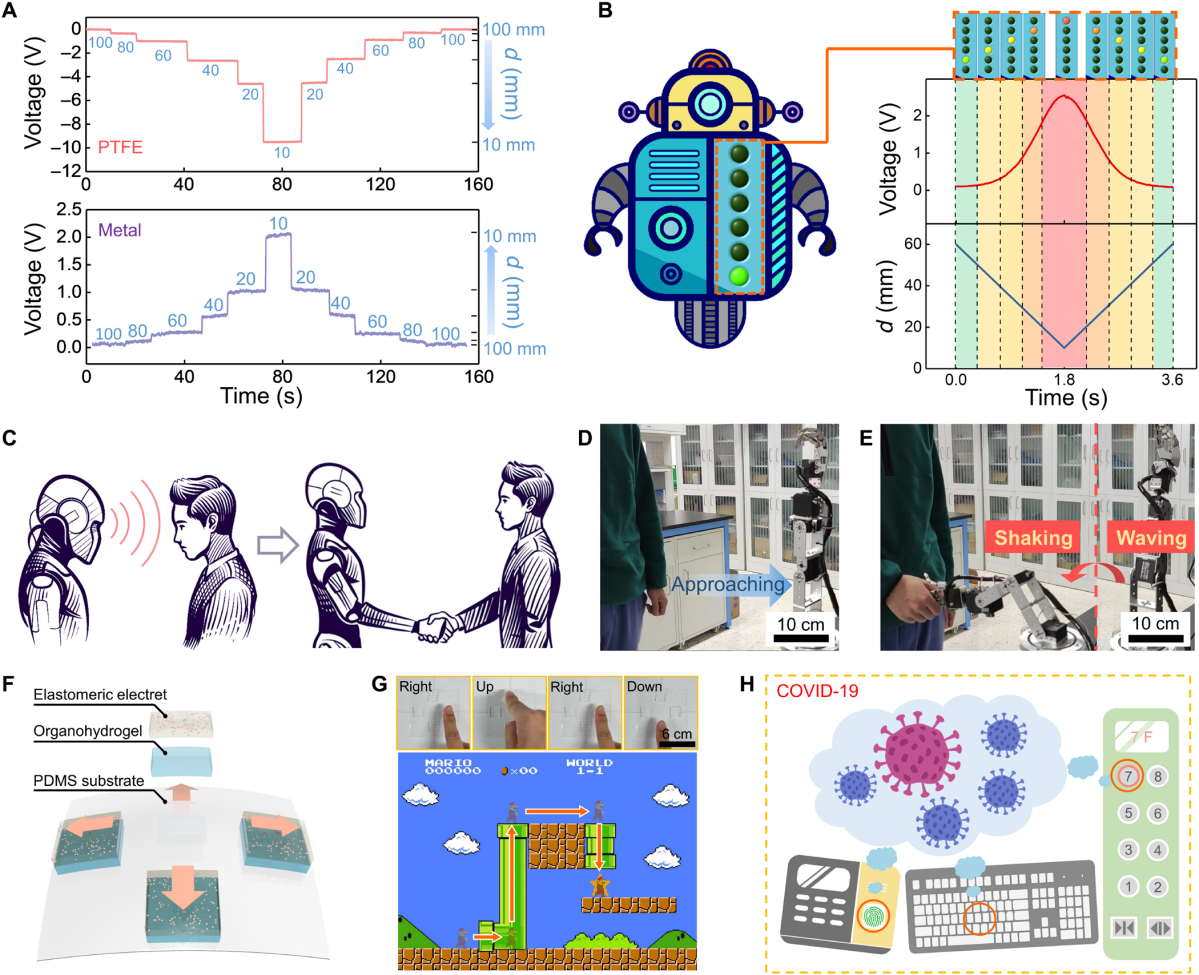
Noncontact HMIs based on the electroreceptor. (**A**) The real-time output response of the electroreceptor to the proximity of the external targets (top: PTFE film and down: metal film). (**B**) The virtual distance alert robot based on the electroreceptor for distance sensing. (**C**) The envisioned scenario of applied the electroreceptor into the intelligent robot system. (**D** and **E**) Demonstration of manipulating robot arm (waving and shaking hands with human) when an adult is approaching. (**F**) The structure of the touchless control pad based on four electroreceptor units. (**G**) Demonstrations of playing Super Mario using our touchless control pad. (**H**) Envisioned application of the touchless control pad to prevent the risk of viruses during the COVID-19 pandemic.

The output of an electroreceptor is related to both the surface charge density and the distance of the target. Nevertheless, the precise distance sensation is possible for a system with determined two relatively approaching objects, as shown by the measured real-time output voltage when targets are approaching (Fig. 3A). Enabled by the sensitive and stable signal response, we then developed a virtual distance alert robot interface based on a customized LabVIEW program (Fig. 3B). The robot is equipped with a series of indicator lights, each of which is switched by a calibrated threshold voltage. Hence, when a target (copper film in our demonstration) approaches, the output voltage of the electroreceptor is monitored in real time, and an appropriate-level indicator light will be turned on when the output voltage reaches the corresponding threshold level. Besides, in this system, when the gap distance between the electroreceptor and the target is less than 30 mm, the indicator light will change to yellow from green; when it further shortens to 20 mm, the light will change to red for intuitive warning. The whole process is highly responsive and fast (movie S2).

For many HMI applications, certain-level voltage signals can trigger the interactions, and precise distance sensation is not required. Novel touchless HMIs were then demonstrated by virtue of the electroreceptor. Figure 3C conceptually demonstrates the envisioned scenario, where a robot equipped with an electroreceptor is able to detect the human approaching and then interacts with human beings using the charges naturally carried on the human body. This expectation is verified by the robotic experiments in Fig. 3 (D and E). Specifically, the signals generated by an approaching human are detected by the electroreceptor and then transmitted to a data acquisition board, which processes the data by a specially written software program (LabVIEW). The program analyzes the signals acquired and then delivers appropriate commands to the robot arm (figs. S13 to S15). As demonstrated, voltage signal increases continuously as a man came closer, and once it reaches a precalibrated threshold, the instruction of waving and shaking hands will be sent to the robot (fig. S15 and movie S3). The threshold voltage can be controlled to adapt to the applications. Despite the different surface charge densities carried by approaching targets, the threshold voltage can be controlled to respond to approaching surfaces with higher or lower surface charge density (fig. S16). The touchless interaction makes the electroreceptor attractive in robotics and HMI field.

Dexterous interactions can also be easily managed by the electroreceptor. We created an intuitive and immersed interaction for manipulating a virtual character. A touchless control pad was fabricated to operate the famous electronic game Super Mario (Fig. 3F). In this system, we integrated four discrete electroreceptor units (size of 1.5 cm by 1.5 cm for each unit) into a PDMS substrate (size of 6 cm by 6 cm) (fig. S17). The pixels of the control pad are divided into four regions, which are responsible for different action triggers, corresponding to moving up, down, left, and right, respectively (figs. S18 and S19). The demo of a user playing the Super Mario game through noncontact activation appears in Fig. 3G and movie S4. In general, touch-based HMIs are high-risk and potential vectors of various bacteria and viruses. The situation is particularly serious during the coronavirus disease 2019 (COVID-19) pandemic. Hence, we envision that HMIs beyond the contact-mode paradigm, together with customized terminal design, will become mainstream interaction means in the future (Fig. 3H).

### Machine learning–aided somatosensation system

**Fig. 4.**
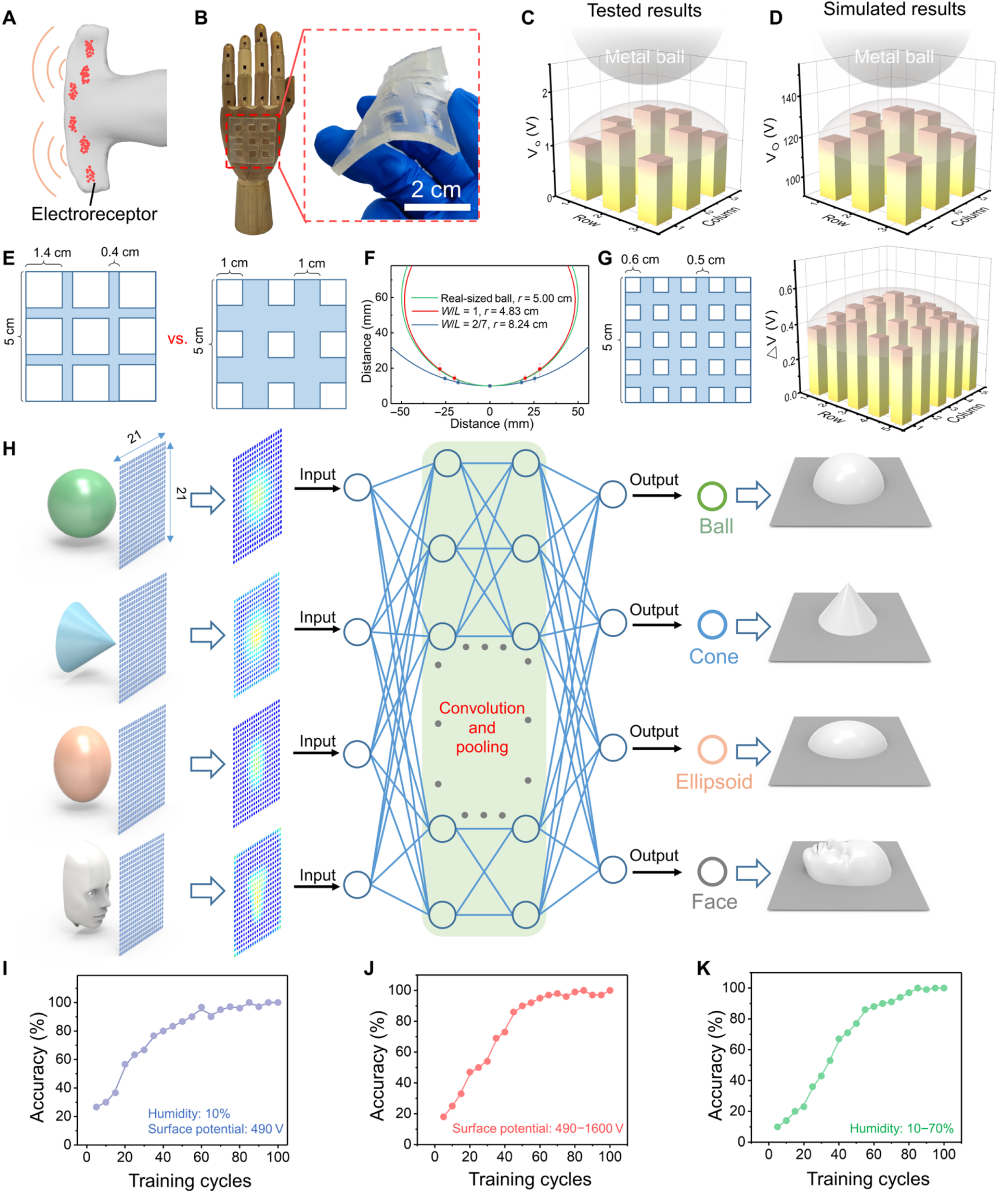
Bioinspired electroreceptor matrix for artificial proximal somatosensory system. (**A**) Electroreceptor networks distributed on the shark’s head. (**B**) The photograph of an electroreceptor matrix made of 3 × 3 units. (**C**) Experimental result and (**D**) simulation result of the 3 × 3 electroreceptor matrix in recognizing the profile of a metal ball. (**E**) The comparison of electroreceptor matrix with different unit sizes and different *W*/*L* ratios (*W*/*L* is 2/7 and 1/1, respectively). (**F**) The comparison of fitted metal ball radius using these two matrices to the real-sized ball. *r*, radius. (**G**) The structure and the voltage profiles of a 5 × 5 electroreceptor matrix with finer unit and the ratio of *W*/*L* = 5/6. (**H**) The 3D object recognition system based on the electroreceptor matrix (21 × 21 units) and the CNN. (**I**) Recognition accuracy of the CNN when humidity was set to 10%, and surface potentials of each sample were set to 490 V. (**J**) Recognition accuracy of the CNN when surface potentials of each sample were changed from 490 to 1600 V. (**K**) Recognition accuracy of the CNN when humidity was changed from 10 to 70%. All samples used in (I) to (K) have unique orientations, distances, and displacements.

Furthermore, we demonstrated a proof of concept that the electroreceptor matrix for three-dimensional (3D) object recognition fused with machine learning algorithms, analogous to a shark’s sensing system (Fig. 4A). We first fabricated a flexible electroreceptor matrix (Fig. 4B), which consists of 3 × 3 square units (unit length *L* = 1.4 cm and adjacent unit space *W* = 0.4 cm). To evaluate the recognition ability of the electroreceptor matrix, we monitored a real-time voltage mapping with a metal ball placed above the electroreceptor matrix at a distance (to bottom) of 10 mm. Results from experiments (Fig. 4C) and simulation (Fig. 4D) were highly consistent, from which a mapping with the highest value in central was distinctly obtained. This is exactly in accordance with the spherical surface, whose center position is the closest to the electroreceptor matrix.

The spatial resolution of the electroreceptor matrix can be improved by optimizing the *W*/*L* ratio (text S2), because it affects the voltage output of each electroreceptor unit due to the inevitable edge effect; i.e., the superposition of electric field induces distortion at the edges. On the basis of our previous theoretical simulation, the edge effect is high at low *W*/*L* ratio (too close distribution of the units), and an optimum *W*/*L* ratio is around 0.7. This effect of *W*/*L* ratio was confirmed by the experimental comparison of two electroreceptor matrices with the same area and 3 × 3 unit number but different unit sizes and *W*/*L* ratios (Fig. 4E and fig. S20). After fitting the measured data, the matrix *W*/*L* = 1/1 shows better accuracy in fitted ball radius (4.83 cm) than matrix *W*/*L* = 2/7 (8.24 cm) and is close to the real-sized ball (5.0 cm) (Fig. 4F). Hence, cautions should be paid to keep a proper *W*/*L* ratio when improving the resolution. We further reduced the unit size and increased the unit numbers to 5 × 5, with the ratio of *W*/*L* = 5/6 (Fig. 4G). The ball surface profile can be obtained by the voltage profile (Fig. 4G), and the fitted ball radius shows even improved accuracy (5.03 cm) (fig. S21). Therefore, it is demonstrated that the 3D profile recognition accuracy and spatial resolution can be improved when designing the matrix with proper *W*/*L* ratio.

In general, devices with high resolution usually require high computing power and complex processing circuit, although they can execute more delicate recognition for different targets. In this case, machine learning algorithms provide an alternative solution to expand more functions with minimized number of sensors (*39*). Here, we constructed an artificial proximal somatosensory system for 3D recognition system through inputting simulation results from electroreceptor matrix into a convolutional neural network (CNN). To obtain a more accurate potential distribution, we further increase the number of the electroreceptor units to 441 (21 × 21). Besides, the potential distribution of the electrostatic receptors from simulation results were converted to 2D images through a “data-to-color” method (i.e., red for higher potential tends and blue for lower potential), so that the CNN could be more convenient to extract the features of the images. In this work, four categories of geometric configuration were selected for the CNN of deep learning: ball, cone, ellipsoid, and human face (Fig. 4H). We established a training dataset and a test dataset to implement the 3D recognition tasks based on the system. The training dataset contains 196 samples that are divided into four categories (fig. S22). Every sample from each category has a unique position and angle. The humidity was set to 10%, and surface potential of each sample was set to 490 V. The test dataset is consisted of four pictures that were never presented to training dataset (fig. S23). The Visual Geometry Group (VGG-16) model is selected as the basic framework of the CNN, because it is advantageous over the feature extraction because of the procession of a smaller convolution kernel. All the units in the CNN are illustrated in fig. S24. It could be found that there are about 1,632,0644 units in the model; therefore, to simplify the calculation, we imported weights in the model from the ImageNet, which were pretrained. When deep learning was started, the recognition accuracy would gradually reach 97% after 75 cycles of training (Fig. 4I). After that, the trained 3D recognition system was tested by the dataset, and the results are shown in figs. S25 to S28. The categories of “ball,” “cone,” “ellipsoid,” and “face” are labeled as “0,” “1,” “2,” and “3,” respectively. If the image of a ball is input into the system, then the output is 0 (fig. S25), which represents the successful recognition of the ball. Similar results are obtained when we input the cone, ellipsoid, and face into the system, respectively (fig. S26 to S28). Further improvements are possible through different classification methods and additional training (*40*).

It is encouraging to find that the 3D profile recognition is possible even when the target surfaces carry different charge densities. The characteristic voltage profiles of electroreceptor matrix are comparable, despite that the absolute voltage values vary with the charge density of targets, as experimentally demonstrated in fig. S29. Deep learning–aided 3D surface profile recognition was then demonstrated even when the target carries different charges and in different shapes. The training dataset of samples that have different surface potentials is demonstrated in fig. S30, and the humidity is set to 10% at the same time. In this condition, the recognition accuracy will gradually reach to 99% after 80 cycles of training (Fig. 4J). The humidity will affect the surface charge densities due to the screen effect as well. As demonstrated in fig. S31, the voltage output of a single electroreceptor was decreased as the humidity is increasing, but when the humidity decrease back to the initial state, the output of electroreceptor will be also recovered. For the electroreceptor matrix, the characteristic voltage profiles are still comparable when the humidity is 70% (fig. S32). On the basis of the experimental results in figs. S31 and S32, a training dataset under different humidity was built (fig. S33). As illustrated in Fig. 4K, after 85 cycles training, the recognition accuracy could gradually reach to 100%. This is verified that the recognition can be implemented in environments with different relative humidity. In terms of temperature, the electrical signals only showed slight increase under −20° to 10°C due to the coagulation of the water vapor (fig. S12). Therefore, the profile recognition accuracy should be not influenced, which is similar to the humidity effects discussed before. One possible limitation is that this strategy might not be applicable to target surface with multiple nonuniform materials. Therefore, this 3D recognition system can open new application fields in next-generation intelligent electronics.

## DISCUSSION

The demands for more versatile human-machine interactions require sensing systems beyond conventional direct contact mode. We take initial inspiration from the shark’s electroreception strategy and fabricate a stretchable transparent artificial electroreceptor to sense approaching targets by detecting the charges that they carry naturally. The artificial electroreceptor is able to encode environmental precontact information into voltage pulses, and various types of targets, including metals, glasses, plastics, polymers, and natural materials, are sensed successfully. HMIs without physical contact are then developed to demonstrate various applications including sense approaching targets, manipulate robot arms, and play computer games. Combined with machine learning algorithms, the feasibility of using electroreceptor matrix to construct an artificial proximal somatosensory system for 3D object recognition is also demonstrated.

Compared with state-of-the-art touchless sensors, such as laser, radio frequency, and ultrasonic sensors, our electroreceptor might be a deficit in distance sensing accuracy; whereas the electroreception is more responsive when reducing the distance, which might be advantageous as the short-distance accuracy could be a limitation for laser or ultrasonic sensors. Furthermore, these unique advantages would make our electroreceptor more competitive in certain applications, including (i) the intrinsic softness, stretchability, transparency, and biocompatibility due to the utilization of elastomer and hydrogel materials; (ii) the negligible power consumption of the sensor as it generates voltage signals from motions by itself; and (iii) the applicability to vast kinds of materials, despite that it is transparent, conductive, magnetic, or not. Therefore, our proposed strategy in realizing electroreception with a soft device is believed to enrich perception dimensions, and we envision that human-interfaced electronics beyond the contact-mode paradigm will become a mainstream interaction means especially in this COVID-19 pandemic.

## MATERIALS AND METHODS

### Materials

*N*,*N*,*N*′,*N*′-tetramethylethylenediamine, *N*,*N*-methylenebisacrylamide, ammonium persulfate, acrylamide, PDMS (Sylgard 184), SiO_2_ nanoparticles, and LiCl were all purchased from Sigma-Aldrich.

### Fabrication of the elastomeric electret

An elastomeric electret was fabricated as follow: (i) SiO_2_ nanoparticles (0.1 g) were first weighed. (ii) The SiO_2_ nanoparticles were subsequently mixed into 10 ml of ethyl alcohol and ultrasonically dispersed for 20 min to form a homogeneous suspension. (iii) The precursor of the PDMS was added into the SiO_2_–ethyl alcohol suspension and then stirred for 10 min. (iv) The mixture was put into an oven with a temperature of 70°C for more than 12 hours to ensure that the ethyl alcohol fully evaporated. (v) The curing agent was added into the mixture and stirred for 5 min. (vi) The mixture of the SiO_2_ nanoparticles and PDMS was poured into a model, which has a hole (400 mm by 400 mm by 0.4 mm) on it. (vii) The model, together with the composite, was put into the oven, and the composite was cured under 70°C for 3 hours. Last, the elastomeric electret film with a thickness of 400 μm contained 2 weight % SiO_2_ nanoparticles that was fabricated.

### Thermal charging process

The schematic diagram and the parameter curve of the thermal charging process are shown in fig. S2A. To conduct the thermal charging process, a pair of fixtures was first built. The fixture consists of two glass plates as substrates and two Cu foils as electrodes. The Cu foils were pasted on the glass plates to serve as electrodes and electrically connected by the Cu wires. The elastomeric electret film was sandwiched between two electrodes. At the beginning of the thermal charging process, the temperature rapidly rose to 100°C through a heating plate. Then the temperature was kept under 100°C for 60 min. Last the heating plate was turned off, and the elastomeric electret was let to naturally cool to room temperature. The strength of the electric field between the electrodes was kept to 3.75 kV/mm through a high-voltage source after the temperature was reached to 100°C.

### Fabrication of the organohydrogel

Acrylamide powder (1.6 g) and 1.2 g of LiCl were added into a mixed solution that consisted of 2 ml of deionized water and 8 ml of EG. Then, 0.01 g of *N*,*N*-methylenebisacrylamide and 0.05 g of ammonium persulfate were dissolved into the mixture above to form a liquid hydrogel. Furthermore, the liquid hydrogel was poured into a culture dish that has a diameter of 30 mm, and *N*,*N*,*N*′,*N*′-tetramethylethylenediamine was subsequently added into the liquid hydrogel. After being placed under room temperature for 1 hour, the organohydrogel was successfully fabricated.

### Calculation of surface charge density of elastomeric electret

The surface charge density herein was calculated through the surface potential of the elastomeric electret. After the thermal charging process, the surface potential of elastomeric electret was first measured by a noncontact electrostatic voltmeter (Monroe, model 279). Then, through the following equation, the surface potential of the elastomeric electret could be calculated

$$\sigma =V\frac{\varepsilon_{r}\varepsilon_{0}}{d}$$ (1)

where σ is the surface charge density, *V* is the surface potential, and ε_r_ and ε_0_ are the relative permittivity of the electret film and the air, respectively. In this work, ε_r_ is measured to be 2.96.

### Fabrication of the 3 × 3 electroreceptor matrix

First, the mixture of the PDMS and SiO_2_ nanoparticles was poured into a mold and followed by a curing process. After peeling off from the mold, an elastomeric electret film with nine holes on was fabricated. The size of each hole is 10 mm by 10 mm, and distances between two adjacent holes are also 10 mm. Then the elastomeric electret film was treated by a thermal charging process. Afterward, the liquid organohydrogel was homogeneously added into nine holes, respectively, and the catalyst was subsequently added to make the organohydrogel electrodes rapidly cured. Last, a layer of very high bond (VHB) tape (3M VHB 9469) was attached to the bottom of the electroreceptor matrix to simply package the electrode.

### Characterization and measurements

A step motor (LinMot E1100) was used to control the distance between the objects and the electroreceptor. The voltage of the electroreceptor was measured through a Keithley 6517 electrometer. To optimize the charge density of elastomeric electret, a contact-separation model triboelectric nanogenerator (TENG) was fabricated, and the elastomeric electret was served as the triboelectrification layer of the TENG. The output of the TENG was also measured by Keithley 6517. A higher output of the TENG represents a larger charge density of the elastomeric electret. The scanning electron microscopy images were obtained from a scanning electron microscope (Hitachi SU8020). The strain-stress tests were conducted by universal materials tester (YL-S71). The electric field during the thermal charging process was provided by a high-voltage polarization instrument (Nanjing Entai Electronic Instruments, ET 2673A). The temperature during the thermal charging process was provided via a heating plate (MS-H280-Pro). To illustrate the applicability of the electroreceptor for different materials, the output of the electroreceptor was measured when 12 different materials approached. During the test, a step motor (LinMot E1100) was still used to control the distance between the objects and the electroreceptor, and the minimum distance was set to 5 mm. All the 12 materials were selected from our laboratory as it is, and their surface charge densities were measured as follows: PTFE, ~33.0 μC/m^2^; Kapton, ~21.0 μC/m^2^; nylon, ~34.2 μC/m^2^; polyethylene terephthalate (PET), ~30.3 μC/m^2^; metal, ~27.4 μC/m^2^; hand, ~11.3 μC/m^2^; wood, ~10.4 μC/m^2^; leaf, ~9.5 μC/m^2^; print paper, ~7.0 μC/m^2^; cotton, ~4.7 μC/m^2^; glass, ~3.6 μC/m^2^; and toilet paper, ~2.0 μC/m^2^. The dielectric constant was measured through an LCR meter (ZM2376). The real-time voltage mapping of the electroreceptor matrix was conducted by a Keithley 6517 electrometer to measure the output of each electroreceptor in sequence. For the ion conductivity test by ac impedance, the area of the sample is 1.77 cm^2^, and the thickness of the sample is 3 mm. The ionic conductivity of the organohydrogel electrode is measured through the ac impedance method (CHI 660).

## Supplementary Materials

### This PDF file includes:

Texts S1 and S2 Figs. S1 to S33 References
