# Supplementary material for: Bioinspired soft electroreceptors for artificial precontact somatosensation
Source: Sci Adv. 2022 May 27;8(21):eabo5201. doi: 10.1126/sciadv.abo5201 (PMC9140963; doi:10.1126/sciadv.abo5201)
Supplement: Supplementary file 1 — Texts S1 and S2 Figs. S1 to S33 References [file sciadv.abo5201_sm.pdf]

Science Advances
AAAS

Supplementary Materials for

Bioinspired soft electroreceptors for artificial precontact somatosensation

Zi Hao Guo *et al.*

Corresponding author: Xiong Pu, puxiong@binn.cas.cn; Zhong Lin Wang, zhong.wang@mse.gatech.edu

Sci. Adv. **8**, eabo5201 (2022)
DOI: 10.1126/sciadv.abo5201

The PDF file includes:

Texts S1 and S2
Figs. S1 to S33
Legends for movies S1 to S4
References

Other Supplementary Material for this manuscript includes the following:

Movies S1 to S4

Supplementary Text 1

1: The establishment of the physical model.

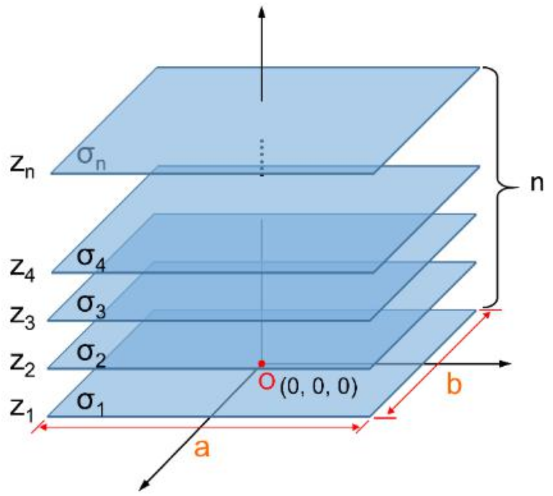

Fig. S1

The physical model of a set of charged finite-sized planes.

To begin with, the physical model of a set of charged finite-sized planes is established (Fig. S1). We assume that there have *n* finite-sized planes and they have the same dimensions *a* and *b* along the *x* and *y* directions. All planes are centered at (x, y)=(0, 0) and located at positions z_1, z_2, …, z_n with the surface charge densities σ_1, σ_2, …, σ_n. The electric potential (ϕ) at an arbitrary point r=(x, y, z) is

$$\phi(x,y,z) = \sum_{i=1}^{n} \int_{-a/2}^{a/2} \int_{-\frac{b}{2}}^{\frac{b}{2}} \frac{\sigma_i dx' dy'}{4\pi\varepsilon(r)\sqrt{(x-x')^2+(y-y')^2+(z-z_i)^2}}$$

$$= \sum_{i=1}^{n} \frac{\sigma_i}{4\pi\varepsilon(r)} \int_{-a/2}^{a/2} \int_{-b/2}^{b/2} \frac{dx' dy'}{\sqrt{(x-x')^2+(y-y')^2+(z-z_i)^2}} \quad (1)$$

Where ε(r) is the permittivity constant of the material at position r.

The electric field (E) is

$$\mathrm{E}(x,y,z) = -\nabla\phi$$

$$= \sum_{i=1}^{n} \frac{\sigma_i}{4\pi\varepsilon(r)} \int_{-a/2}^{a/2} \int_{-b/2}^{b/2} \frac{dx' dy'}{(x-x')^2+(y-y')^2+(z-z_i)^2} \frac{(x-x', y-y', z-z_i)}{\sqrt{(x-x')^2+(y-y')^2+(z-z_i)^2}} \quad (2)$$

When integrating over the x' and y' directions, where x=y=0, the electric field along the z direction is

$$E(0,0,z) = \sum_{i=1}^{n} \frac{\sigma_i(z-z_i)}{4\pi\varepsilon(r)} \int_{-a/2}^{a/2}\int_{-b/2}^{b/2} \frac{dx'dy'}{\left(x'^2+y'^2+(z-z_i)^2\right)^{3/2}}$$

$$= \sum_{i=1}^{n} \frac{\sigma_i(z-z_i)}{\pi\varepsilon(r)(z-z_i)} arctan\left(\frac{ab}{4(z-z_i)\sqrt{\left(\frac{a}{2}\right)^2+\left(\frac{b}{2}\right)^2+(z-z_i)^2}}\right) \quad (3)$$

2: The simulation of the electroreceptor

According to the physical model above and Fig. 2a, we assume that there is a space coordinates system located on the center of organo-hydrogel electrode. The equivalent charge planes of the ionic electrode, the elastomeric electret and external object are located at z_1, z_2, and z_3 positions with the same size, respectively. The electric potential of the electrode at the position (0, 0, z_1) is

$$\phi(0,0,z_1) = \frac{\sigma_3}{\pi\varepsilon_1}\int_{0}^{\infty} f(z')\,dz' - \frac{\sigma_2}{\pi\varepsilon_1}\int_{z_1-z_2}^{\infty} f(z')\,dz' + \frac{\sigma_1}{\pi\varepsilon_1}\int_{z_1-z_3}^{\infty} f(z')\,dz' \quad (4)$$

$$f(z') = arctan\left(\frac{ab}{4(z')\sqrt{\left(\frac{a}{2}\right)^2+\left(\frac{b}{2}\right)^2+z'^2}}\right) \quad (5)$$

Where σ_1, σ_2 and σ_3 are the surface charge density of external object, the elastomeric electret and the ionic electrode, respectively; z represents the separation distance between the electroreceptor and external object; ε_1 is the permittivity of the elastomeric electret.

Under the open-circuit condition, σ_3=0, therefore the open-circuit potential value of the electrode could be simplified as:

$$\phi(0,0,z_1)_{OC} = -\frac{\sigma_2}{\pi\varepsilon_1}\int_{z_1-z_2}^{\infty} f(z')\,dz' + \frac{\sigma_1}{\pi\varepsilon_1}\int_{z_1-z_3}^{\infty} f(z')\,dz' \quad (6)$$

The numerical calculation results of this model will be commendably consistent with the finite element simulation results of COMSOL. This has been adequately proven by our previous works (*41, 42*). The analytic calculations of the equations above are extremely complicated. Therefore, in this work, COMSOL simulation was selected to visually reveal the variation tendency of the electrode potential when an object approaches the electroreceptor.

Supplementary Text 2

The optimization of electroreceptor matrix for higher resolution

The resolution of the electroreceptor array here could be defined as the number of electroreceptor pixels per unit area. Obviously, the higher density of the pixels leads to a higher resolution and better profile recognition. However, there exists the edge effect for the electrorecetpor. The electric field lines of the electroreceptor units interfere with each other according to the superposition theorem, which hence affects the voltage output of every single electroreceptor. We previously carried out work on array triboelectric nanogenerators (TENG), and studied the edge effects of electrostatic filed theoretically (*41*). According to this work, the edge effect is highly related to the geometry structure and distribution of each unit (*41*).

Assuming the length of each square unit is represented by L, and the space between two adjacent units is W. Then, the W/L ratio has a direct effect on the electroreceptor unit's output. The output voltage of the electroreceptor unit will be decreased as reducing W/L ratio. Clearly, a small W/L implies a small distance between the units, such that the electric fields originating from the edges of units interfere significantly with each other, thereby generating distortion electric field distributions. This is easy to be understood that the edge effect will be suppressed when the units are sparsely distributed. This trend has been demonstrated in our last study as shown in the above figures. On the other hand, as W/L increases, the spacial resolution will be reduced, as the too-sparsely distributed arrays will be unable to identify complex geometric shapes. Therefore, there seems to be a trade-off between the dense unit distribution and high voltage resolution. However, the spatial resolution can still be improved by increasing the unit numbers and reducing the unit sizes, but cautions should be paid, that the W/L ratio should be kept to be around a certain optimum value to not exacerbate the edge distortion effect. According to the previous study, the optimized W/L ratio is around 0.7 (*41*).

To verify whether the resolution can be improved by geometry optimization and to prove the proposed technology that is available for the "deep learning", some necessary experiments have been carried out by varying the geometry and layout of the electroreveptor arrays:

Take the 3×3 array as an example. We firstly designed and fabricated two 3×3 electroreceptor arrays whose total working area is 5×5 cm^2. Two kinds of W/L ratios were adopted. The first one: W = 1 cm and L = 1 cm, W/L=1; the second one: W = 0.4 cm and L = 1.4 cm, W/L=2/7 (Fig. 4E). We used the same metal ball with the same radius, r= 5 cm, to approach the two electroreceptor array at the same height of 1 cm, respectively. It can be found that although the unit voltage of the array W/L=2/7 is larger than that of the one W/L=1, the voltage distinction between each unit becomes smaller. This means the voltage resolution is improved. By using the height distance-voltage relationship obtained in Fig. 2F, we calculated and fitted the ball radius using the two arrays. The results showed that the larger W/L=1 ratio leads to a more accurate fitted ball radius of 4.84 cm, much improved from the 8.24 cm when W/L is 2/7 (Fig. 4F).

Then, we further developed a 5×5 electroreceptor array with more and finer pixels. The W/L of which was set to W = 0.5 cm and L = 0.6 cm, W/L=5/6 (Fig. 4G). The fitted ball diameter has even further improved accuracy of 5.03 cm (Fig. S19).

Therefore, these data showed clearly that, by optimizing the W/L ratio, higher resolution can be achieved with finer and more electroreceptor units.

The ball radius was fitted by the measured data of the electroreceptor matrix through the following method: the voltage of each electroreceptor was experimentally recorded, and the distance of the ball surface right on top of each electroreceptor can be then calculated by fitted curves in Fig. 2F, where the target distance-voltage relationship can be fitted with experimental data. Due to the rotational symmetry of the ball shape, the 9 distance values of 3×3 electroreceptor units can be projected and averaged into 5 points on the projected circle. Then the ball radius can be calculated and compared with real ball size, for evaluating the measurement accuracy.

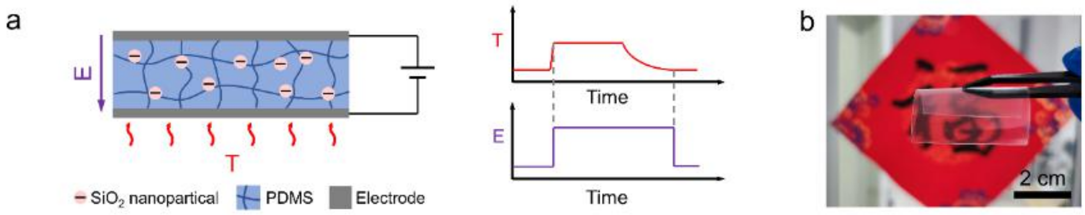

Fig. S2.

Thermal-charging process of the elastomeric electret. (a) The schematic diagram and the parameter curve of the thermal charging process. (b) The photograph of the prepared elastomeric electret film which exhibits excellent flexibility and transparency of the elastomeric electret.

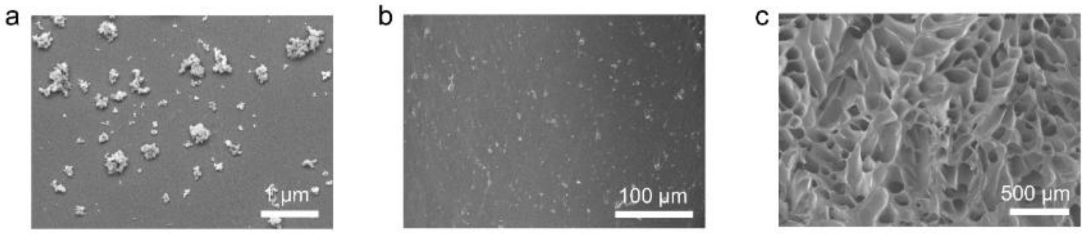

Fig. S3.

SEM characterization of the electret. SEM images of (a) SiO_2 nanoparticles, (b) cross-section of elastomeric electret and (c) organo-hydrogel, from which homogeneous dispersion of the SiO_2 NPs in PDMS matrix and the porous structure of organo-hydrogel could be clearly observed.

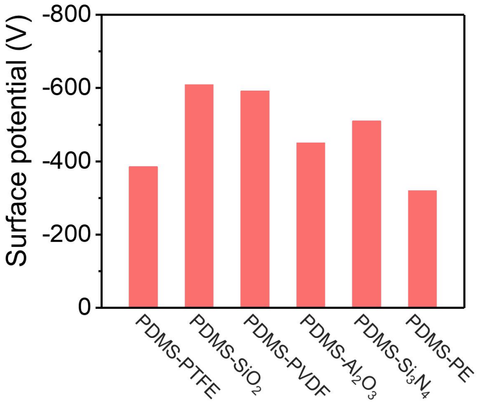

Fig. S4.

Surface potentials of different composite electret after thermal charging process.

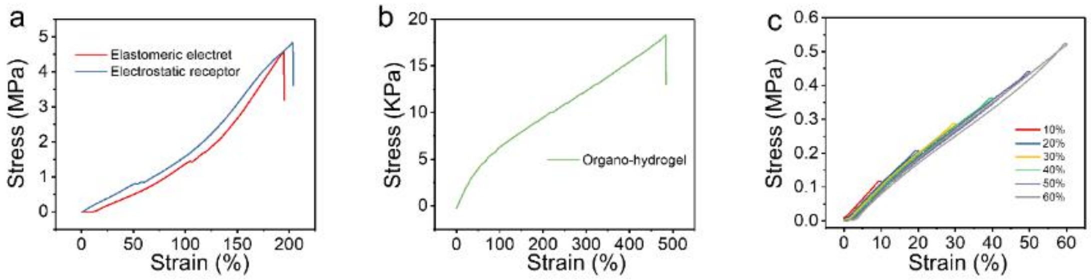

Fig. S5.

The elastic properties of the electret. The strain-stress curves of (a) elastomeric electret and electroreceptor, (b) organo-hydrogel. (c) The strain cycles of electroreceptor. Experimental results reveal that the electroreceptor shows great mechanical stretchability, which can accommodate tensile strain exceeds 190% and shows no residual strains after strain cycle tests.

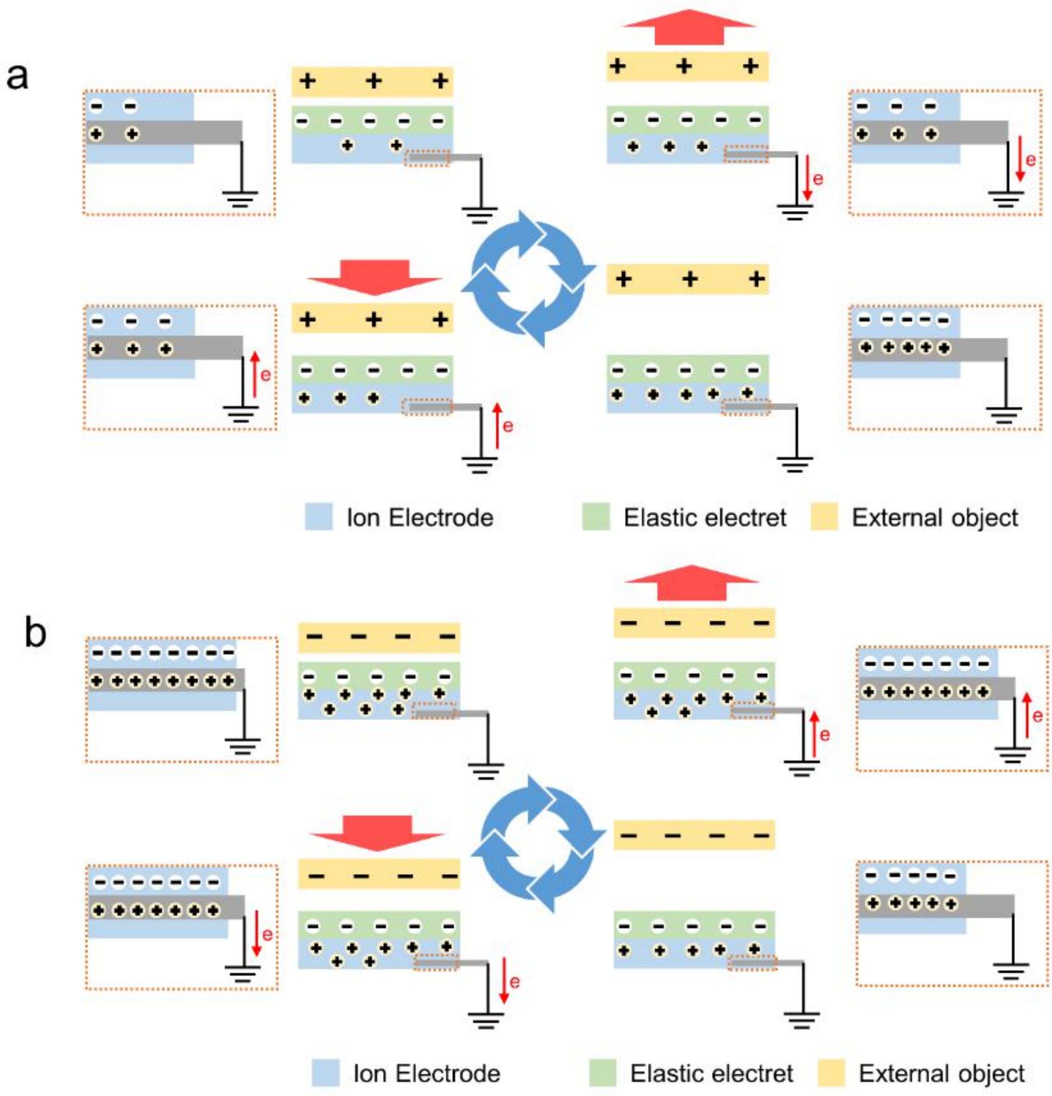

Fig. S6.

Working principle of the electroreceptor. Situation when an object carried (a) positive charges or (b) negative charges approaching.

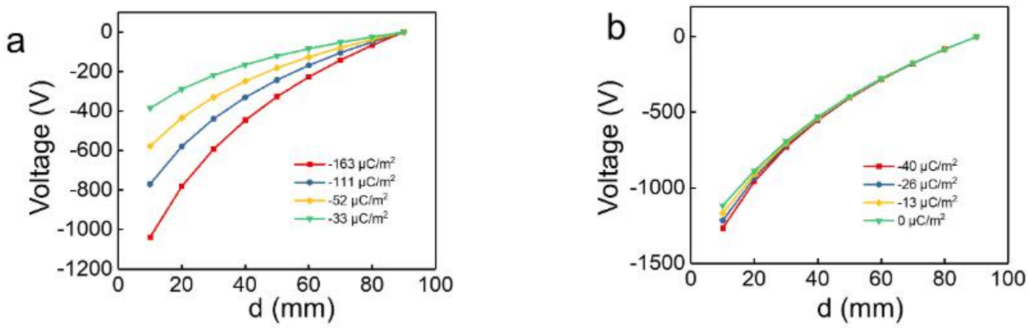

Fig. S7.

The simulation results of COMSOL. (a) the relationship between the output voltage of electroreceptor and σ_1 (the surface charge density of object), (b) the relationship between the output voltage of electroreceptor and σ_2 (the surface charge density of elastomeric electret).

The simulation results reveals that the distance-sensitivity and voltage absolute value increase dramatically with the charge density σ_1 of the approaching surface, but are insensitive to the charge density of the electret σ_2. These results are consistent with the experiment results in Fig. 2D-E. As for metal object, when increasing the surface charge density of elastomeric electret, the surface charge density of metal will be gradually increased due to the electrostatic induction. Therefore, the variation of the voltage output in Fig. 2F is similar to Fig.2D.

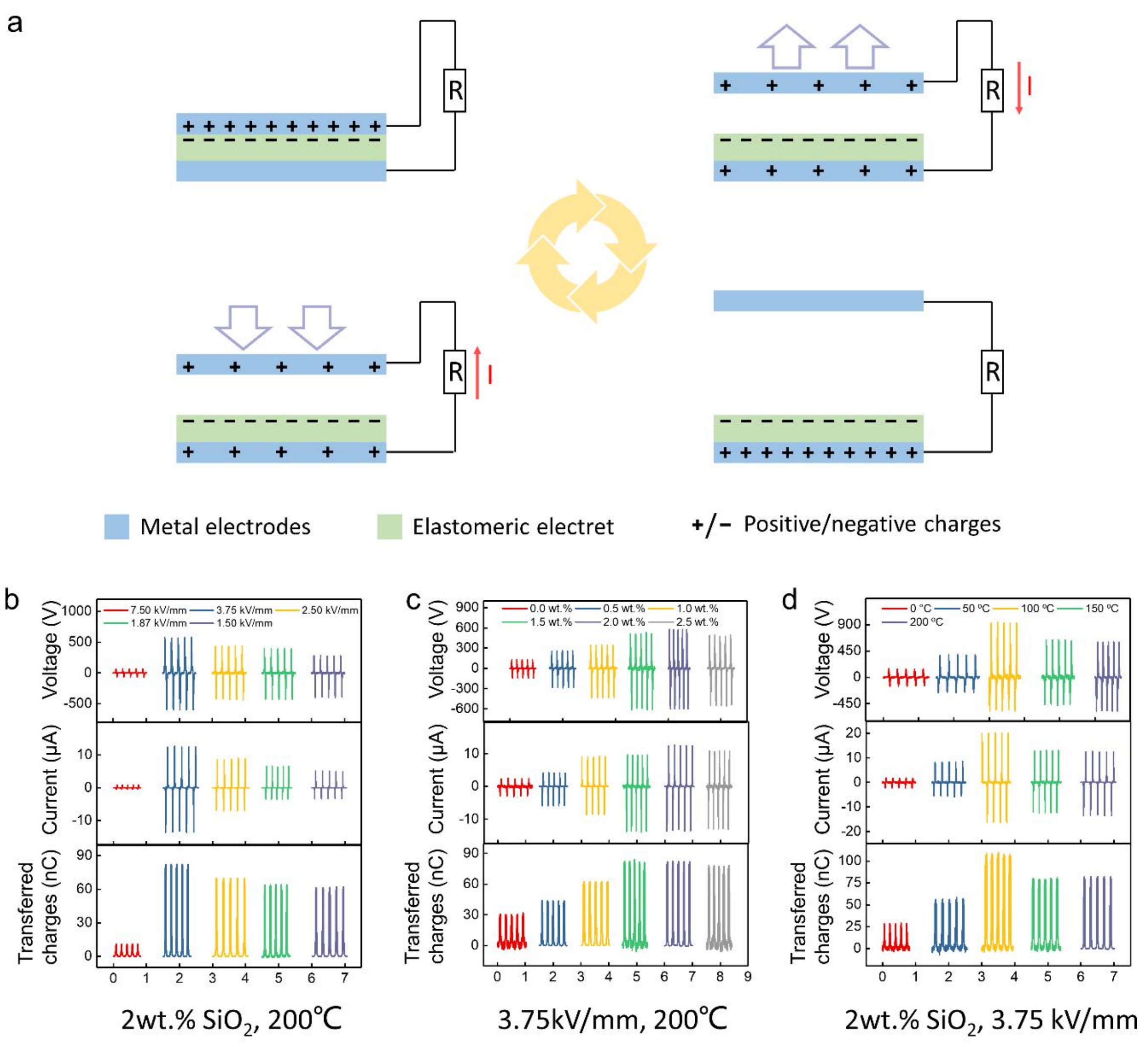

Fig. S8.

The optimization of the charge density of the elastomeric electret. (a) The working principle of the TENG. (b) The output variations of the TENGs when control the charging temperature and the SiO_2 fraction of the elastomeric electret, and change the charging electric field intensity of the elastomeric electret. (c) The output variations of the TENGs when control the charging temperature and the charging electric field intensity of the elastomeric electret, and change the SiO_2 fraction of the elastomeric electret. (d) The output variations of the TENGs when control the SiO_2 fraction and the charging electric field intensity of the elastomeric electret, and change the charging temperature the elastomeric electret.

To optimize the charge density of elastomeric electret, a contact-separation model triboelectric nanogenerator (TENG) was fabricated and the elastomeric electret was served as

the triboelectrification layer of the TENG. The output of the TENG was also measured by Keithley 6517. According to the working principle of the TENG (Fig. S8(a)), the output of the TENG will be comprehensively affected by the equivalent surface charge density due to the electrostatic induction effect. Therefore, a higher output of the TENG represents a larger charge density of the elastomeric electret. Therefore, a higher output of the TENG represents a larger charge density of the elastomeric electret.

From Fig. S8(b), it could be found that when control the charging temperature and the SiO_2 fraction of the elastomeric electret, the output of the TENG was increased with the growth of charging electric field intensity. However, when the electric field intensity was increased to 7.5 kV/mm, the output was extremely decreased. It could be caused by the breakdown of the elastomeric electret under a high electric field intensity. Therefore, the best charging electric field intensity is 3.75 kV/mm.

Fig. S8(c) shows that as the SiO_2 fraction increasing the output of the TENG was increased. However, when the SiO_2 fraction exceed 2 wt.%, the output was slightly decreased. It could be caused by the aggregation of the SiO_2 nano-particles. Therefore, the optimal SiO_2 fraction should be 2 wt.%.

The relationship between the charging temperature and the output of the TENG is illustrated in Fig. S8(d). It could be seen that the output of the TENG was increasing when the charging temperature increased, but when the temperature exceed 100 ℃, the output was slightly decreased. A higher charging temperature would aggravate the thermal motion of the molecules. Under this condition, the negative charges will have more difficulties to be captured by SiO_2 nano-particles. Hence, the optimal charging temperature should be 100 ℃

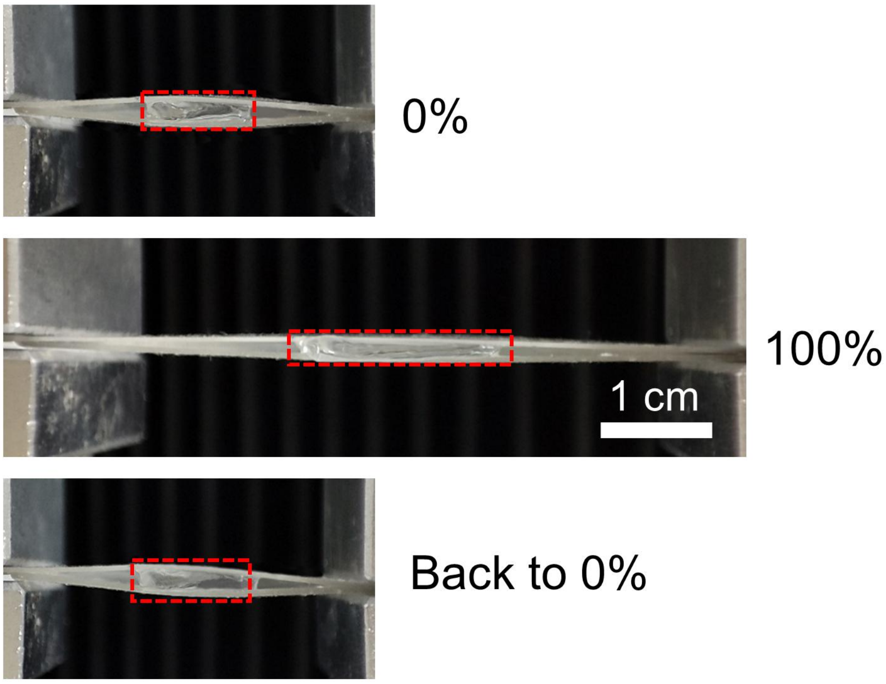

Fig. S9.

Side-view photographs of electroreceptor during the stretch process.

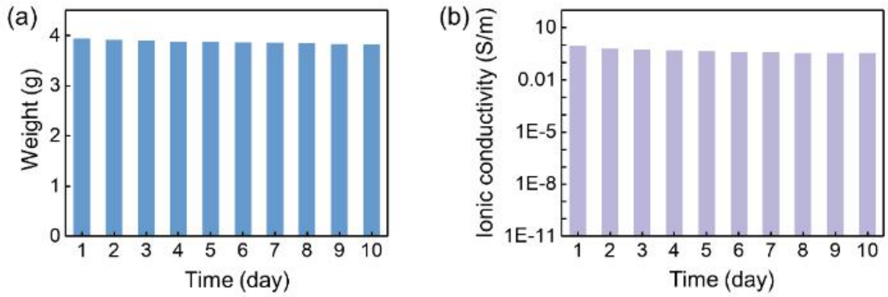

Fig. S10.

The durability of the ionic electrode. The variation of (a) weight and (b) ionic conductivity of the organo-hydrogel electrode in 10 days under room temperature.

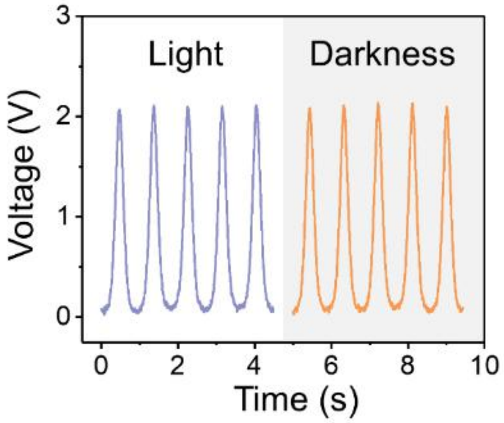

Fig. S11.

The output of the electroreceptor under light or darkness conditions

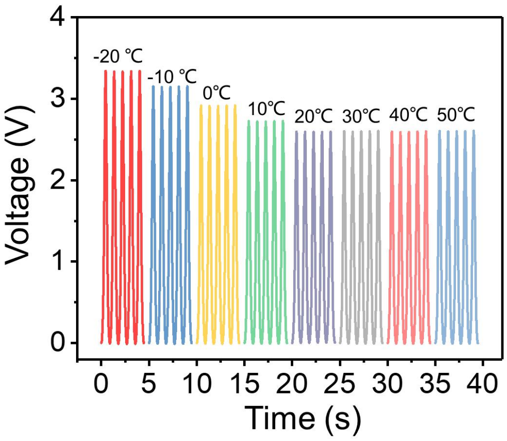

Fig. S12.

The output of the electroreceptor under -20~ 50 ℃.

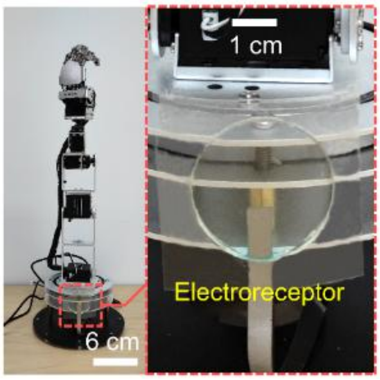

Fig. S13.

The photograph of the electroreceptor integrated robot arm.

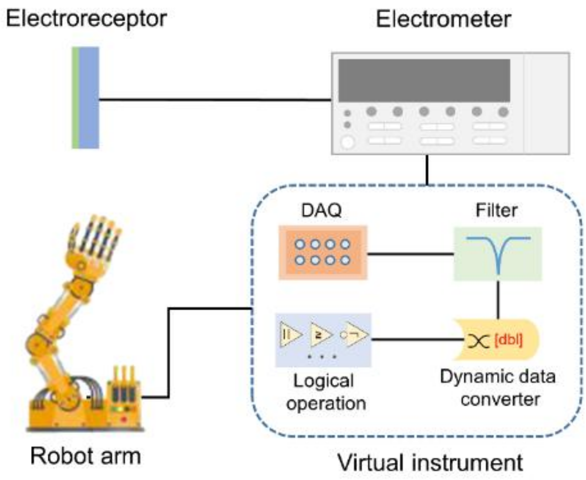

Fig. S14.

The structure of the distance perception system.

The signals generated by human approaching are detected by the electroreceptor and then transmitted to a virtual instrument which is constructed by a specially written software program (LabVIEW) and consisted of a Data Acquisition (DAQ) module, a filter, a dynamic data converter and logical operation module. The virtual instrument analyzes signals acquired and then delivers appropriate commands to the robot arm.

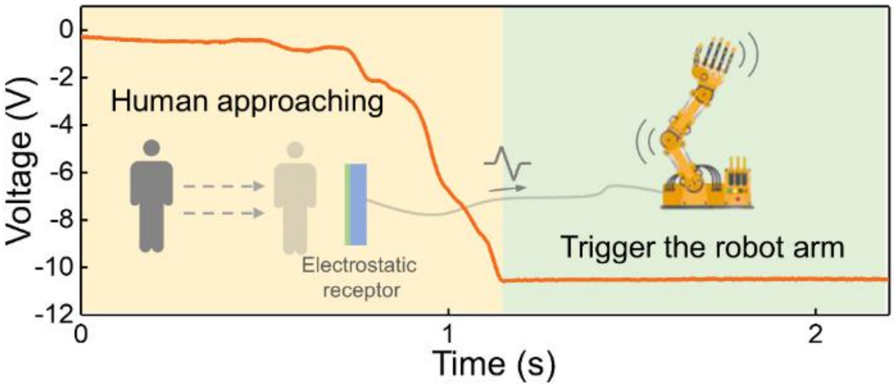

Fig. S15.

An example as the human-machine interface. The voltage signal that accumulated by electrometer when the human approaching the electroreceptor and trigger the robot arm.

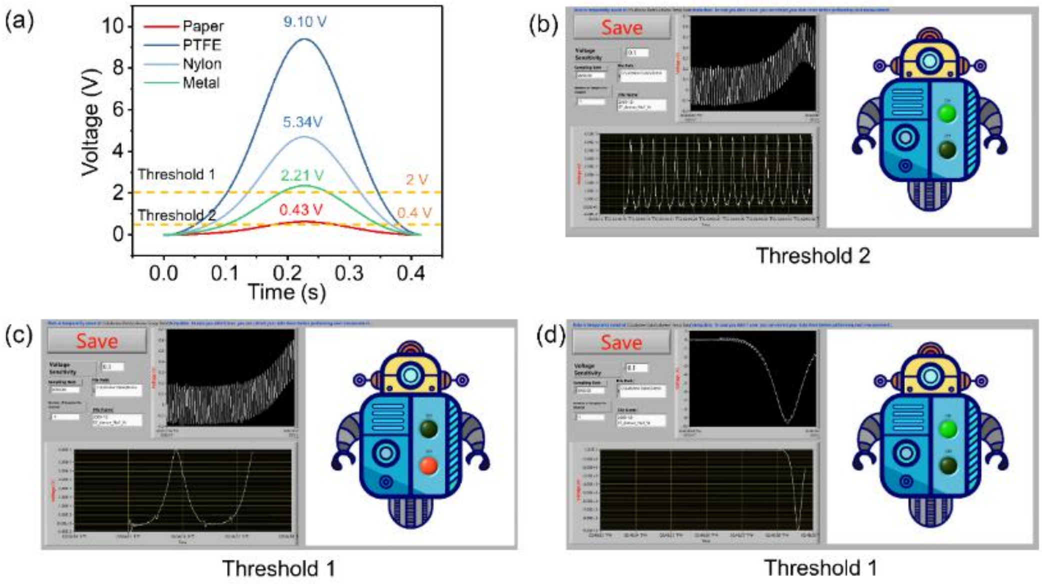

Fig. S16.

The selection of the threshold voltage. (a) The output of the electroreceptor when materials with different surface charge density approaching. (b) the virtual robot with threshold 2 (0.4 V) was triggered by approaching paper. (c) the virtual robot with threshold 1 (2 V) failed to be triggered by approaching paper. (d) the virtual robot with threshold 1 (2 V) was triggered by approaching PTFE.

As shown in Fig. S14, the voltage output of the electroreceptor was rise to 9.1 V and 0.43 V respectively when PTFE and paper approach. If the threshold voltage was set to 2 V, the virtual robot could be triggered by PTFE, but couldn't be triggered by paper (Fig. S14 (c-d)). However, if we set the threshold voltage to 0.4 V, the virtual robot could be triggered by both PTFE and paper (Fig. S14 (b)).

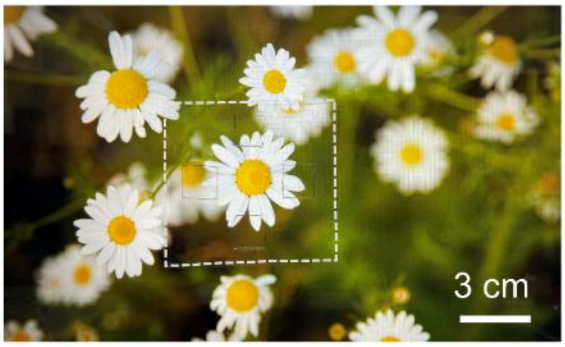

Fig. S17.

The photograph of the touchless keyboard.

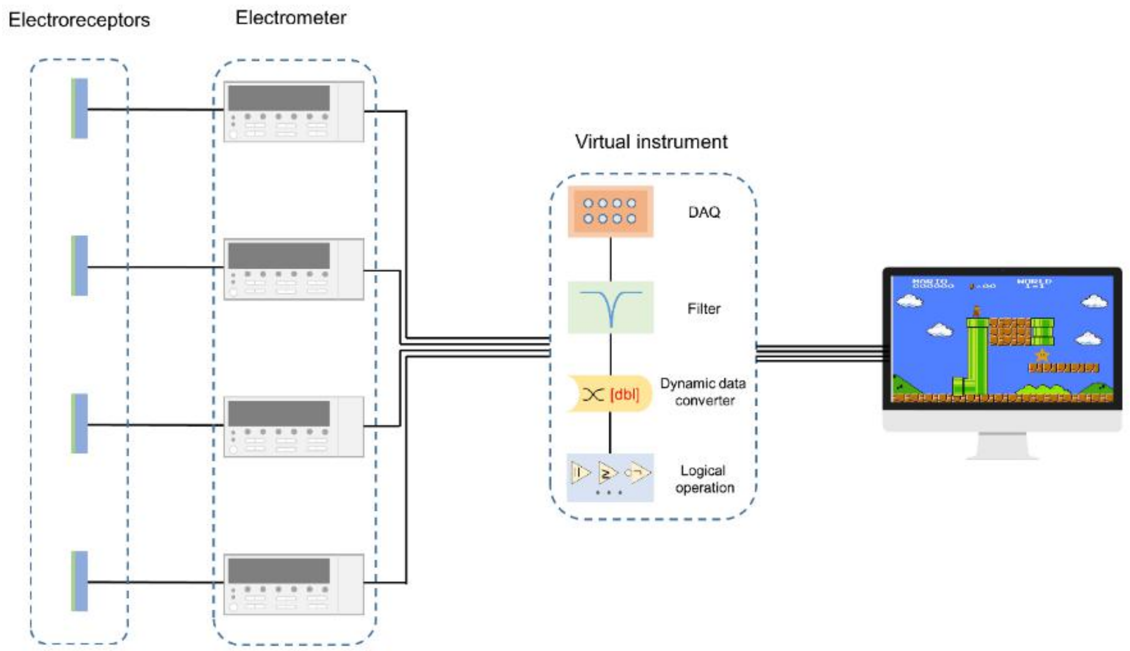

Fig. S18.

The schematic diagram of the multichannel data acquisition system.

Signals of up, down, left, and right regions on the touchless pad are collected by a multichannel data acquisition system illustrated in Fig. S13. The multichannel data acquisition system contains four channels (corresponding to up, down, left, and right regions), each channel has an electroreceptor and an electrometer to perceive the approaching of the finger. Then, collected signals will be passed to a virtual instrument based on LabVIEW to control the movement of Super Mario.

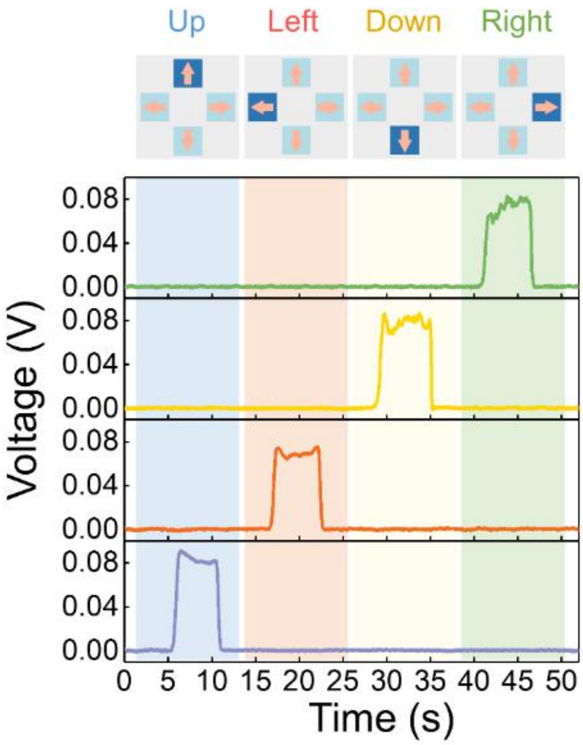

Fig. S19.

Signals from "up", "down", "right" and "left" channels.

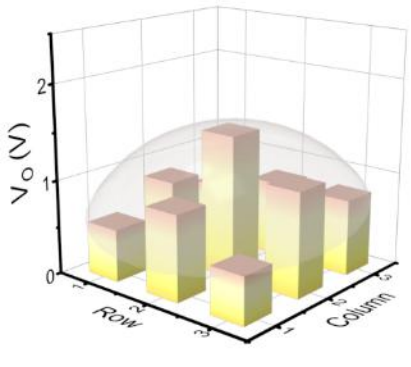

Fig. S20.

The output of the 3×3 electroreceptor matrix with the W/L=1.

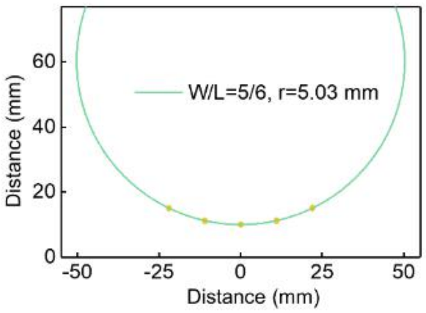

Fig. S21.

The fitted ball radius using the 5×5 electroreceptor matrix with W/L=5/6.

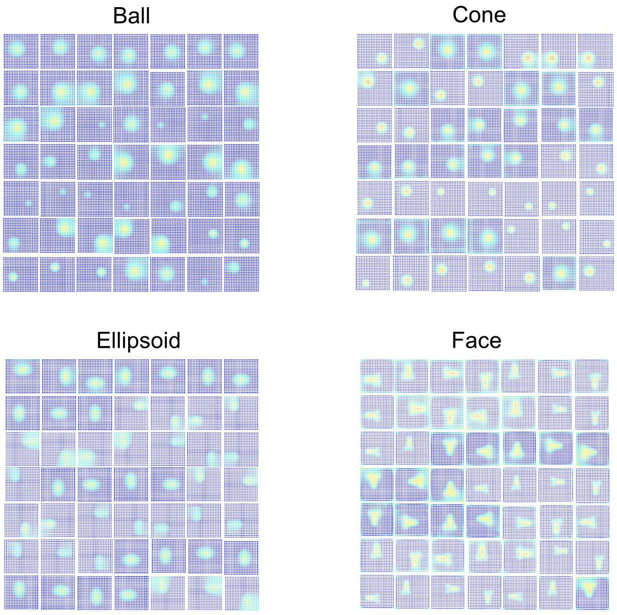

Fig. S22.

The training dataset of the “Ball”, “Cone”, “Ellipsoid” and “Face” category.

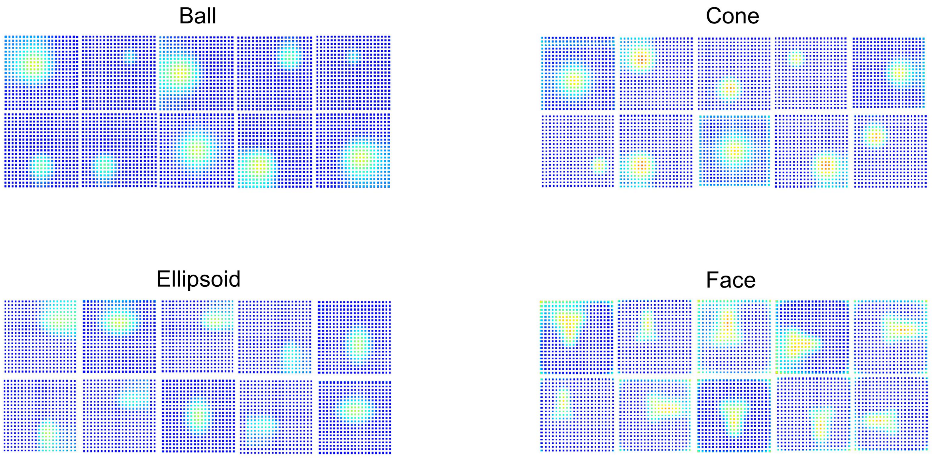

Fig. S23.

The test dataset. The test dataset of (a) “ball”, (b) “cone”, (c) “ellipsoid”, (d) “face” category.

Layer (type)	Output Shape	Param #
input_9 (InputLayer)	[(None, 240, 240, 3)]	0
block1_conv1 (Conv2D)	(None, 240, 240, 64)	1792
block1_conv2 (Conv2D)	(None, 240, 240, 64)	36928
block1_pool (MaxPooling2D)	(None, 120, 120, 64)	0
block2_conv1 (Conv2D)	(None, 120, 120, 128)	73856
block2_conv2 (Conv2D)	(None, 120, 120, 128)	147584
block2_pool (MaxPooling2D)	(None, 60, 60, 128)	0
block3_conv1 (Conv2D)	(None, 60, 60, 256)	295168
block3_conv2 (Conv2D)	(None, 60, 60, 256)	590080
block3_conv3 (Conv2D)	(None, 60, 60, 256)	590080
block3_pool (MaxPooling2D)	(None, 30, 30, 256)	0
block4_conv1 (Conv2D)	(None, 30, 30, 512)	1180160
block4_conv2 (Conv2D)	(None, 30, 30, 512)	2359808
block4_conv3 (Conv2D)	(None, 30, 30, 512)	2359808
block4_pool (MaxPooling2D)	(None, 15, 15, 512)	0
block5_conv1 (Conv2D)	(None, 15, 15, 512)	2359808
block5_conv2 (Conv2D)	(None, 15, 15, 512)	2359808
block5_conv3 (Conv2D)	(None, 15, 15, 512)	2359808
block5_pool (MaxPooling2D)	(None, 7, 7, 512)	0
flatten_8 (Flatten)	(None, 25088)	0
dense_16 (Dense)	(None, 64)	1605696
dense_17 (Dense)	(None, 4)	260

Total params: 16,320,644
Trainable params: 16,320,644
Non-trainable params: 0

Fig. S24.

Units of the CNN network.

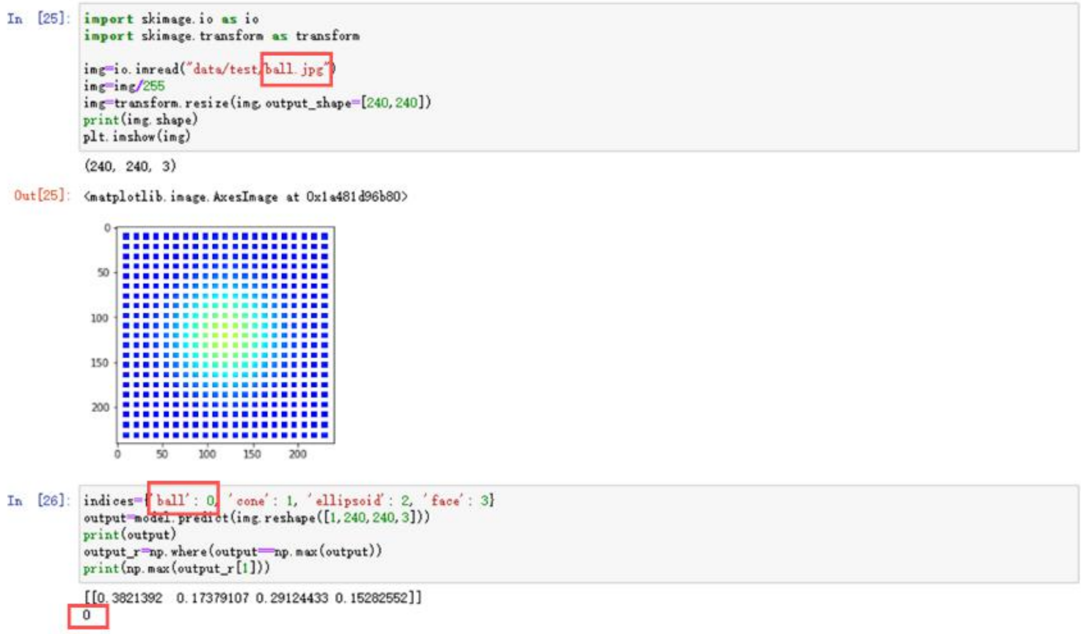

Fig. S25.

Test result when the image of “ball” was input into the trained skin vision system.

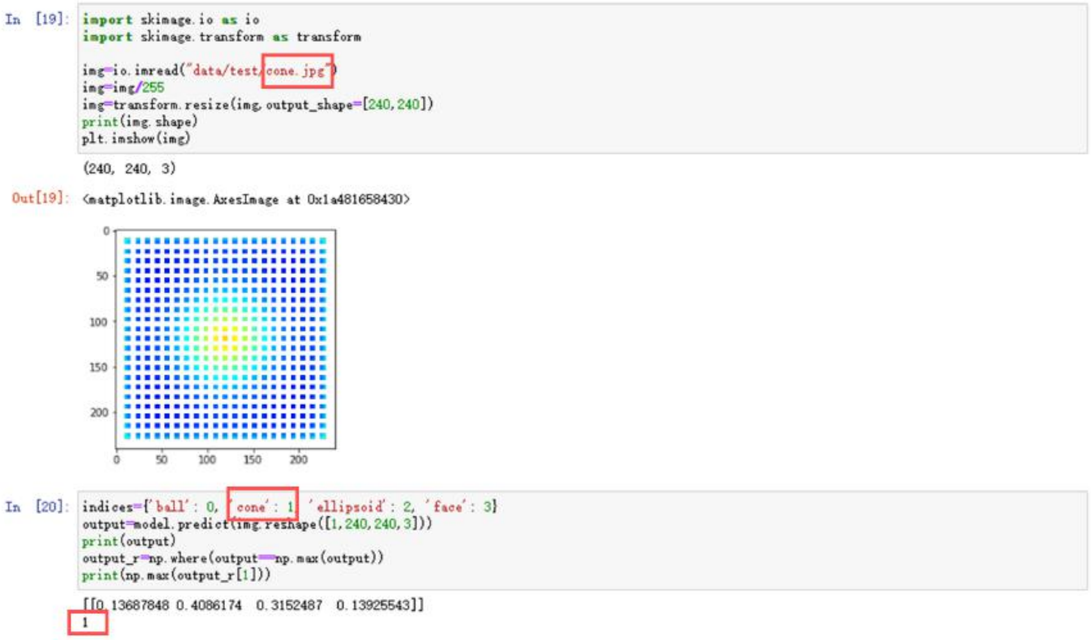

Fig. S26.

Test result when the image of "cone" was input into the trained skin vision system.

```
In [23]: import skimage.io as io
         import skimage.transform as transform

         img=io.imread("data/test/ellipsoid.jpg")
         img=img/255
         img=transform.resize(img,output_shape=[240,240])
         print(img.shape)
         plt.imshow(img)

(240, 240, 3)

Out[23]: <matplotlib.image.AxesImage at 0x1a481bda9a0>
```

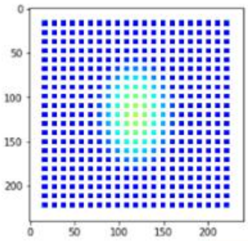


```
In [24]: indices={'ball': 0, 'cone': 1, 'ellipsoid': 2, 'face': 3}
         output=model.predict(img.reshape([1,240,240,3]))
         print(output)
         output_r=np.where(output==np.max(output))
         print(np.max(output_r[1]))

[[0.13734296 0.20592913 0.5529719  0.10375609]]
2
```

Fig. S27.

Test result when the image of "ellipsoid" was input into the trained skin vision system.

```
In [21]: import skimage.io as io
         import skimage.transform as transform

         img=io.imread("data/test/face.jpg")
         img=img/255
         img=transform.resize(img,output_shape=[240,240])
         print(img.shape)
         plt.imshow(img)

(240, 240, 3)

Out[21]: <matplotlib.image.AxesImage at 0x1a481a0e730>
```

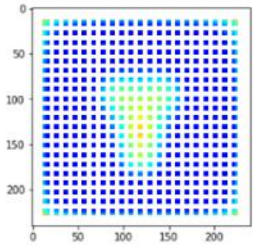


```
In [22]: indices={'ball': 0, 'cone': 1, 'ellipsoid': 2, 'face': 3}
         output=model.predict(img.reshape([1,240,240,3]))
         print(output)
         output_r=np.where(output==np.max(output))
         print(np.max(output_r[1]))

[[0.173405   0.13982493 0.199728   0.48704207]]
3
```

Fig. S28.

Test result when the image of "face" was input into the trained skin vision system.

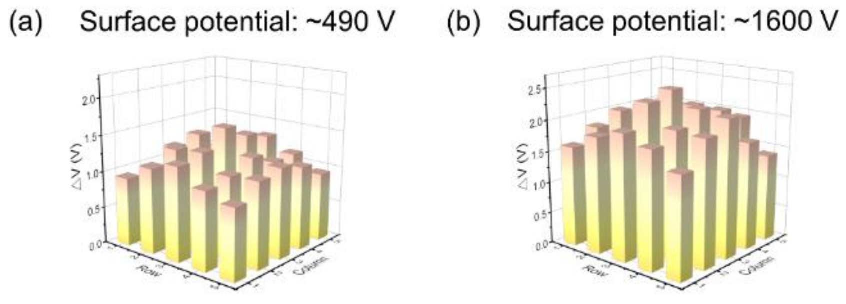

Fig. S29.

The experimental voltage output of 5×5 electroreceptor array when detecting PTFE ball targets with different surface charge density.

Although the output of each electroreceptor array is different, the characteristic profiles (sphere) are still the comparable.

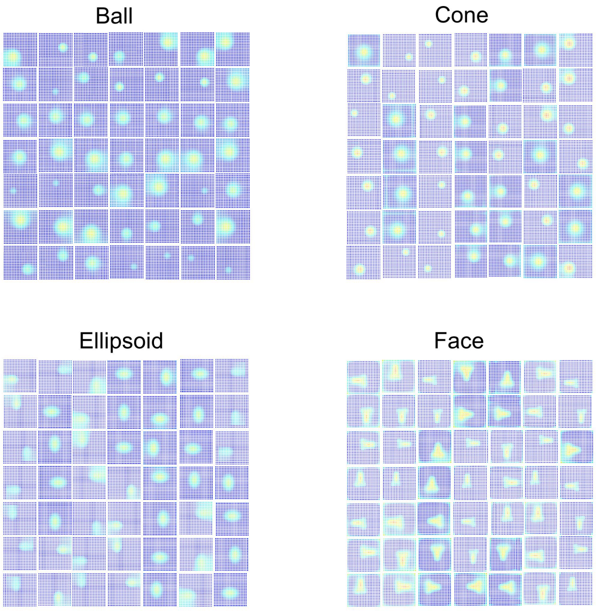

Fig. S30.

The training dataset of the “ball”, “cone”, “ellipsoid” and “face” category. Surface potential of each samples was changed from 490 to 1600 V

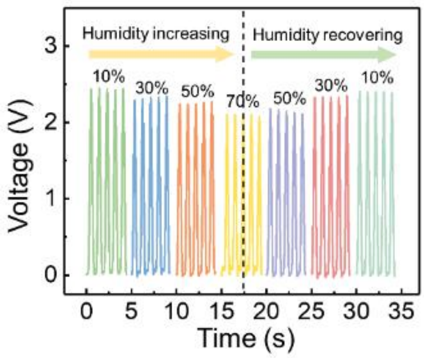

Fig. S31.

The voltage output of a single electroreceptor when exposed in different humidity.

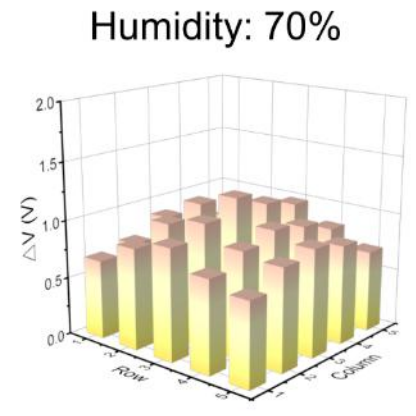

Fig. S32.

The experimental voltage output of 5×5 electroreceptor array when detecting ball targets in 70% humidity environment.

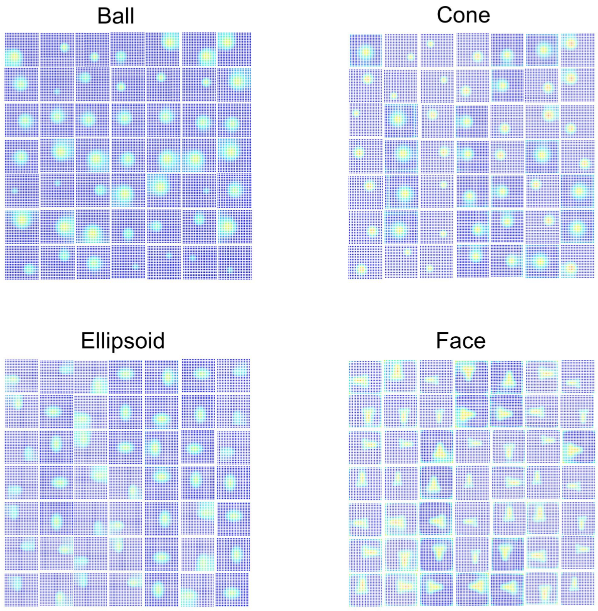

Fig. S33.

The training dataset of the "ball", "cone", "ellipsoid" and "face" category under different humidity. Here, four different surface charge densities are selected to mimic the four humidity conditions (10%, 30%, 50% and 70%), as the humidity will affect the surface charge densities.

Movie S1.

Simulation results. Part 1: the simulation of metal approaching the electroreceptor. Part 2: the simulation of PTFE approaching the electroreceptor

Movie S2.

Electroreceptor for virtual distance alert robot.

Movie S3.

Interacting with a robot arm through the electroreceptor.

Movie S4.

Electroreceptor array for human-machine interface.

REFERENCES AND NOTES

1. W. G. Bircher, A. S. Morgan, A. M. Dollar, Complex manipulation with a simple robotic hand through contact breaking and caging. *Sci. Robot.* **6**, eabd2666 (2021).

2. H. C. Zhao, K. O'Brien, S. Li, R. F. Shepherd, Optoelectronically innervated soft prosthetic hand via stretchable optical waveguides. *Sci. Robot.* **1**, eaai7529 (2016).

3. C. M. Boutry, M. Negre, M. Jorda, O. Vardoulis, A. Chortos, O. Khatib, Z. N. Bao, A hierarchically patterned, bioinspired e-skin able to detect the direction of applied pressure for robotics. *Sci. Robot.* **3**, eaau6914 (2018).

4. I. You, D. G. Mackanic, N. Matsuhisa, J. Kang, J. Kwon, L. Beker, J. Mun, W. Suh, T. Y. Kim, J. B. Tok, Z. Bao, U. Jeong, Artificial multimodal receptors based on ion relaxation dynamics. *Science* **370**, 961–965 (2020).

5. B. C. K. Tee, A. Chortos, A. Berndt, A. K. Nguyen, A. Tom, A. McGuire, Z. L. C. Lin, K. Tien, W. G. Bae, H. L. Wang, P. Mei, H. H. Chou, B. X. Cui, K. Deisseroth, T. N. Ng, Z. N. Bao, A skin-inspired organic digital mechanoreceptor. *Science* **350**, 313–316 (2015).

6. M. Kanik, S. Orguc, G. Varnavides, J. Kim, T. Benavides, D. Gonzalez, T. Akintilo, C. C. Tasan, A. P. Chandrakasan, Y. Fink, P. Anikeeva, Strain-programmable fiber-based artificial muscle. *Science* **365**, 145–150 (2019).

7. P. D. Marasco, J. S. Hebert, J. W. Sensinger, D. T. Beckler, Z. C. Thumser, A. W. Shehata, H. E. Williams, K. R. Wilson, Neurorobotic fusion of prosthetic touch, kinesthesia, and movement in bionic upper limbs promotes intrinsic brain behaviors. *Sci. Robot.* **6**, eabf3368 (2021).

8. L. E. Osborn, A. Dragomir, J. L. Betthauser, C. L. Hunt, H. H. Nguyen, R. R. Kaliki, N. V. Thakor, Prosthesis with neuromorphic multilayered e-dermis perceives touch and pain. *Sci. Robot.* **3**, eaat3818 (2018).

9. J. Liang, Y. Wu, J. K. Yim, H. Chen, Z. Miao, H. Liu, Y. Liu, Y. Liu, D. Wang, W. Qiu, Z. Shao, M. Zhang, X. Wang, J. Zhong, L. Lin, Electrostatic footpads enable agile insect-scale soft robots with trajectory control. *Sci. Robot.* **6**, eabe7906 (2021).

10. G. Y. Gu, J. Zou, R. K. Zhao, X. H. Zhao, X. Y. Zhu, Soft wall-climbing robots. *Sci. Robot.* **3**, eaat2874 (2018).

11. Y. C. Wu, J. K. Yim, J. M. Liang, Z. C. Shao, M. J. Qi, J. W. Zhong, Z. H. Luo, X. J. Yan, M. Zhang, X. H. Wang, R. S. Fearing, R. J. Full, L. W. Lin, Insect-scale fast moving and ultrarobust soft robot. *Sci. Robot.* **4**, eaax1594 (2019).

12. C. C. Kim, H. H. Lee, K. H. Oh, J. Y. Sun, Highly stretchable, transparent ionic touch panel. *Science* **353**, 682–687 (2016).

13. S. H. Byun, J. Y. Sim, Z. A. Zhou, J. Lee, R. Qazi, M. C. Walicki, K. E. Parker, M. P. Haney, S. H. Choi, A. Shon, G. B. Gereau, J. Bilbily, S. Li, Y. H. Liu, W. H. Yeo, J. G. McCall, J. L. Xiao, J. W.

Jeong, Mechanically transformative electronics, sensors, and implantable devices. *Sci. Adv.* **5**, eaay0418 (2019).

14. N. Papadopoulos, W. M. Qiu, M. Ameys, S. Smout, M. Willegems, F. Deroo, J. L. van der Steen, A. J. Kronemeijer, M. Dehouwer, A. Mityashin, R. Gehlhaar, K. Myny, Touchscreen tags based on thin-film electronics for the Internet of Everything. *Nat. Electron.* **2**, 606–611 (2019).
15. X. Yu, Z. Xie, Y. Yu, J. Lee, A. Vazquez-Guardado, H. Luan, J. Ruban, X. Ning, A. Akhtar, D. Li, B. Ji, Y. Liu, R. Sun, J. Cao, Q. Huo, Y. Zhong, C. Lee, S. Kim, P. Gutruf, C. Zhang, Y. Xue, Q. Guo, A. Chempakasseril, P. Tian, W. Lu, J. Jeong, Y. Yu, J. Cornman, C. Tan, B. Kim, K. Lee, X. Feng, Y. Huang, J. A. Rogers, Skin-integrated wireless haptic interfaces for virtual and augmented reality. *Nature* **575**, 473–479 (2019).
16. Y. X. Shi, F. Wang, J. W. Tian, S. Y. Li, E. G. Fu, J. H. Nie, R. Lei, Y. F. Ding, X. Y. Chen, Z. L. Wang, Self-powered electro-tactile system for virtual tactile experiences. *Sci. Adv.* **7**, eabe2943 (2021).
17. M. L. Zhu, Z. D. Sun, Z. X. Zhang, Q. F. Shi, T. Y. Y. He, H. C. Liu, T. Chen, C. K. Lee, Haptic-feedback smart glove as a creative human-machine interface (HMI) for virtual/augmented reality applications. *Sci. Adv.* **6**, eaaz8693 (2020).
18. L. Lu, C. Jiang, G. Hu, J. Liu, B. Yang, Flexible noncontact sensing for human-machine interaction. *Adv. Mater.* **33**, e2100218 (2021).
19. J. Ge, X. Wang, M. Drack, O. Volkov, M. Liang, G. S. C. Bermudez, R. Illing, C. A. Wang, S. Q. Zhou, J. Fassbender, M. Kaltenbrunner, D. Makarov, A bimodal soft electronic skin for tactile and touchless interaction in real time. *Nat. Commun.* **10**, 4405 (2019).
20. H. C. Guo, Y. J. Tan, G. Chen, Z. F. Wang, G. J. Susanto, H. H. See, Z. J. Yang, Z. W. Lim, L. Yang, B. C. K. Tee, Artificially innervated self-healing foams as synthetic piezo-impedance sensor skins. *Nat. Commun.* **11**, 5747 (2020).
21. Y. J. Zhang, T. H. Tao, A bioinspired wireless epidermal photoreceptor for artificial skin vision. *Adv. Funct. Mater.* **30**, 2000381 (2020).
22. G. A. Howland, P. B. Dixon, J. C. Howell, Photon-counting compressive sensing laser radar for 3D imaging. *Appl. Optics* **50**, 5917–5920 (2011).
23. T. Kim, D.-H. Kang, S. Shim, M. Im, B. K. Seo, H. Kim, B. C. Lee, Versatile low-cost volumetric 3D ultrasound imaging using gimbal-assisted distance sensors and an inertial measurement unit. *Sensors* **20**, 6613 (2020).
24. M. Wang, Z. Yan, T. Wang, P. Q. Cai, S. Y. Gao, Y. Zeng, C. J. Wan, H. Wang, L. Pan, J. C. Yu, S. W. Pan, K. He, J. Lu, X. D. Chen, Gesture recognition using a bioinspired learning architecture that integrates visual data with somatosensory data from stretchable sensors. *Nat. Electron.* **3**, 563–570 (2020).

25. D. J. Schlegel, D. P. Finkbeiner, M. Davis, Maps of dust infrared emission for use in estimation of reddening and cosmic microwave background radiation foregrounds. *Astrophys. J.* **500**, 525–553 (1998).

26. D. Rodrigues-Bigaton, A. V. Dibai, A. C. D. Costa, A. C. Packer, E. M. de Castro, Accuracy and reliability of infrared thermography in the diagnosis of arthralgia in women with temporomandibular disorder. *J. Manipulative Physiol. Ther.* **36**, 253–258 (2013).

27. M. Guix, R. Mestre, T. Patino, M. De Corato, J. Fuentes, G. Zarpellon, S. Sanchez, Biohybrid soft robots with self-stimulating skeletons. *Sci. Robot.* **6**, eabe7577 (2021).

28. M. Liu, X. Pu, C. Jiang, T. Liu, X. Huang, L. Chen, C. Du, J. Sun, W. Hu, Z. L. Wang, Large-area all-textile pressure sensors for monitoring human motion and physiological signals. *Adv. Mater.* **29**, 1703700 (2017).

29. R. M. Kempster, S. P. Collin, Electrosensory pore distribution and feeding in the megamouth shark *Megachasma pelagios* (Lamniformes: Megachasmidae). *Aquat. Biol.* **11**, 225–228 (2011).

30. N. W. Bellono, D. B. Leitch, D. Julius, Molecular basis of ancestral vertebrate electroreception. *Nature* **543**, 391–396 (2017).

31. A. J. Kalmijn, Electric and magnetic-field detection in elasmobranch fishes. *Science* **218**, 916–918 (1982).

32. S. Zhang, Y. Wang, X. Yao, P. Le Floch, X. Yang, J. Liu, Z. Suo, Stretchable electrets: Nanoparticle-elastomer composites. *Nano. Lett.* **20**, 4580–4587 (2020).

33. X. Pu, M. M. Liu, X. Y. Chen, J. M. Sun, C. H. Du, Y. Zhang, J. Y. Zhai, W. G. Hu, Z. L. Wang, Ultrastretchable, transparent triboelectric nanogenerator as electronic skin for biomechanical energy harvesting and tactile sensing. *Sci. Adv.* **3**, e1700015 (2017).

34. H. T. Baytekin, A. Z. Patashinski, M. Branicki, B. Baytekin, S. Soh, B. A. Grzybowski, The mosaic of surface charge in contact electrification. *Science* **333**, 308–312 (2011).

35. M. M. Apodaca, P. J. Wesson, K. J. M. Bishop, M. A. Ratner, B. A. Grzybowski, Contact electrification between identical materials. *Angew. Chem. Int. Ed.* **49**, 946–949 (2010).

36. Z. L. Wang, A. C. Wang, On the origin of contact-electrification. *Mater. Today* **30**, 34–51 (2019).

37. T. Liu, M. Liu, S. Dou, J. Sun, Z. Cong, C. Jiang, C. Du, X. Pu, W. Hu, Z. L. Wang, Triboelectric-nanogenerator-based soft energy-harvesting skin enabled by toughly bonded elastomer/hydrogel hybrids. *ACS Nano* **12**, 2818–2826 (2018).

38. P. Zhang, W. Guo, Z. H. Guo, Y. Ma, L. Gao, Z. Cong, X. J. Zhao, L. Qiao, X. Pu, Z. L. Wang, Dynamically crosslinked dry ion-conducting elastomers for soft iontronics. *Adv. Mater.* **33**, e2101396 (2021).

39. J. H. Han, K. M. Bae, S. K. Hong, H. Park, J.-H. Kwak, H. S. Wang, D. J. Joe, J. H. Park, Y. H. Jung, S. Hur, C. D. Yoo, K. J. Lee, Machine learning-based self-powered acoustic sensor for speaker recognition. *Nano Energy* **53**, 658–665 (2018).

40. Y. Liu, J. J. Norton, R. Qazi, Z. Zou, K. R. Ammann, H. Liu, L. Yan, P. L. Tran, K. I. Jang, J. W. Lee, D. Zhang, K. A. Kilian, S. H. Jung, T. Bretl, J. Xiao, M. J. Slepian, Y. Huang, J. W. Jeong, J. A. Rogers, Epidermal mechano-acoustic sensing electronics for cardiovascular diagnostics and human-machine interfaces. *Sci. Adv.* **2**, e1601185 (2016).

41. J. J. Shao, Y. Yang, O. Yang, J. Wang, M. Willatzen, Z. L. Wang, Designing rules and optimization of triboelectric nanogenerator arrays. *Adv. Energy Mater.* **11**, 2100065 (2021).

42. J. J. Shao, M. Willatzen, Y. J. Shi, Z. L. Wang, 3D mathematical model of contact-separation and single-electrode mode triboelectric nanogenerators. *Nano Energy* **60**, 630–640 (2019).
